# Isomers of Terpyridine as Ligands in Coordination Polymers and Networks Containing Zinc(II) and Cadmium(II)

**DOI:** 10.3390/molecules26113110

**Published:** 2021-05-23

**Authors:** Catherine E. Housecroft, Edwin C. Constable

**Affiliations:** Department of Chemistry, University of Basel, BPR 1096, Mattenstrasse 24a, CH-4058 Basel, Switzerland; edwin.constable@unibas.ch

**Keywords:** 2,2′:6′,2″-terpyridine, 3,2′:6′,3″-terpyridine, 4,2′:6′,4″-terpyridine, coordination polymer, coordination network, zinc(II), cadmium(II)

## Abstract

The use of divergent 4,2′:6′,4″- and 3,2′:6′,3″-terpyridine ligands as linkers and/or nodes in extended coordination assemblies has gained in popularity over the last decade. However, there is also a range of coordination polymers which feature 2,2′:6′,2″-terpyridine metal-binding domains. Of the remaining 45 isomers of terpyridine, few have been utilized in extended coordination arrays. Here, we provide an overview of coordination polymers and networks containing isomers of terpyridine and either zinc(II) and cadmium(II). Although the motivation for investigations of many of these systems is their luminescent behavior, we have chosen to focus mainly on structural details, and we assess to what extent assemblies are reproducible. We also consider cases where there is structural evidence for competitive product formation. A point that emerges is the lack of systematic investigations.

## 1. Introduction

**Scheme 1:**
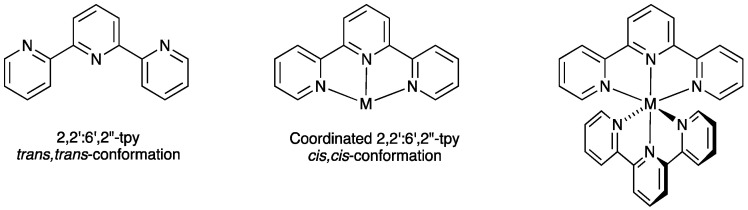
Structure of 2,2′:6′,2″-terpyridine in the free ligand (left) and in a bis-chelate mode, and a typical {M(2,2′:6′,2″-tpy)_2_} domain containing an octahedral metal center.

**Scheme 2:**
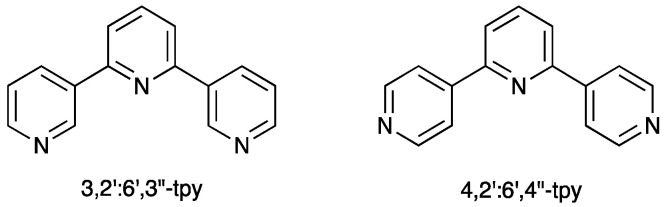
The structures of 3,2′:6′,3″-terpyridine and 4,2′:6′,4″-terpyridine.

**Scheme 3:**
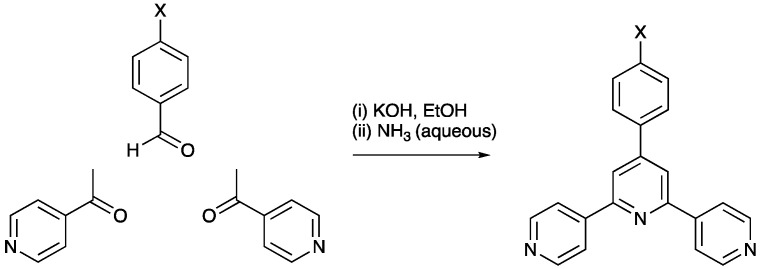
Use of the Hanan one-pot strategy [7] to synthesize a 4′-functionalized 4,2′:6′,4″-tpy. The method can be adapted by using 2-acetylpyridine or 3-acetylpyridine precursors to prepare derivatives of 2,2′:6′,2″-tpy or 3,2′:6′,3″-tpy.

The archetypal terpyridine (tpy) is 2,2′:6′,2″-tpy (Scheme 1) which, as undergraduates learn, prefers a terdentate, bis-chelating mode of coordination to a metal ion. A conformational change from *s-trans,s-trans* to *s-cis*,*s-cis* accompanies metal binding (Scheme 1), and mononuclear complexes containing {M(2,2′:6′,2″-tpy)_2_} (Scheme 1) or {M(2,2′:6′,2″-tpy)X*_n_*} domains are ubiquitous [1,2,3,4]. Two points are often missed in discussions of terpyridine coordination chemistry. The first is that metal coordination compounds in which 2,2′:6′,2″-tpy ligands exhibit hypodentate modes are well represented [5]. The second is that, while 2,2′:6′,2″-tpy is indeed the most studied isomer of terpyridine, another 47 isomers can be drawn (Figure S1 in the Supporting Material). In terms of synthesis, some are certainly far more accessible than others. For example, the Kröhnke [6] and one-pot Hanan [7] strategies allow straightforward routes to symmetrical (with respect to the equivalence of the outer pyridine rings) compounds including 3,2′:6′,3″-tpy and 4,2′:6′,4″-tpy (Scheme 2), albeit with occasional examples where cyclic products are favored [8]. These synthetic routes also permit functionalization of the tpy unit, in particular, at the 4′-position in 2,2′:6′,2″-tpy, 3,2′:6′,3″-tpy and 4,2′:6′,4″-tpy as illustrated in Scheme 3 for the introduction of an aryl group.

**Figure 1:**
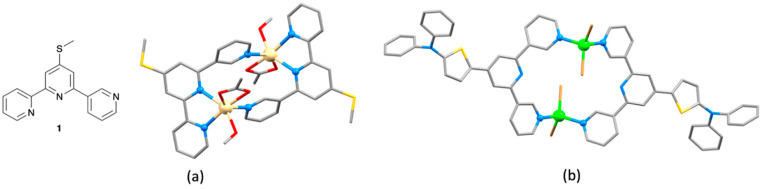
(**a**) The structure of ligand **1** (a derivative of 2,2′:6′,3″-tpy) and the metallocycle [Cd_2_(**1**)_2_(MeOH)_2_(O_2_CMe)_2_]^2+^, structurally characterized as the [PF_6_]^–^ salt (CSD refcode REFFEN). (**b**) An example of a 4′-functionalized 3,2′:6′,3″-tpy in conformation **C** acting as a convergent ligand in [Zn_2_Br_4_L_2_] where L = *N,N*-diphenyl-5-(3,2′:6′,3′′-terpyridin-4′-yl)thiophen-2-amine (CSD refcode ELANIQ).

**Scheme 4:**
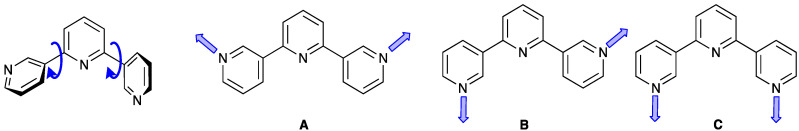
Some isomers of tpy are conformationally flexible, for example, rotation about the inter-ring C–C bonds in 3,2′:6′,3″-tpy leads to three planar conformers.

This review concerns coordination polymers and networks, and the latter are conveniently considered in terms of combinations of organic linkers and metal nodes, organic nodes and metal linkers, or organic nodes and metal nodes. The coordination geometries of the metal centers, combined with the vectorial properties of the ligand donor sites, are key starting points in the design of an assembly. Inspection of Figure S1 quickly leads to the conclusion that (organic synthetic aspects aside), the choice of an isomer of terpyridine is fundamental to selecting building blocks suitable for a polymeric rather than a discrete molecular assembly. For example, 2,2′:6′,3″-tpy (Figure S1) offers a bidentate, chelating site and a remote monodentate site that direct the assembly of a metallomacrocycle rather than a coordination polymer in the reaction of cadmium(II) acetate with ligand **1** (Figure 1) in MeOH to give [Cd_2_(**1**)_2_(MeOH)_2_(O_2_CMe)_2_]^2+^ (Figure 1a) [9]. Some isomers of tpy are conformationally flexible, and this is illustrated in Scheme 4 for 3,2′:6′,3″-tpy for which three planar limiting conformations exist. The ligand conformation can be switched in the solid-state structure by introducing different substituents [10], and this impacts the structures of coordination polymers containing such ligands [11,12,13]. Note that while conformations **A** and **B** in Scheme 4 are divergent, conformation **C** is convergent and often leads to discrete molecular complexes, for example, the dizinc(II) macrocycle [14] shown in Figure 1b. 

In this review, we focus on coordination polymers and networks that contain zinc(II) and cadmium(II) in combination with isomers of terpyridine. A comprehensive survey of compounds that have been structurally characterized is presented and the content is based upon searches of the Cambridge Structural Database (CSD) [15] made using CSD version 2020.3.1 [16] and ConQuest version 2020.3.1 [16]. We have chosen to exclude detailed discussion of assemblies including co-ligands which function as building blocks. However, we note that co-ligands are one means of extending the dimensionalities of assemblies. We also draw attention to cases where a potential co-ligand added to the reaction mixture (usually under solvothermal conditions) appears to act as a template rather than being incorporated into the coordination assembly. The review is organized according to terpyridine isomer, and the functional groups that the ligand carries, and whether the latter are coordinatively innocent or not. Structural diagrams in this review have been drawn using Mercury 2020.3.1 [17] with coordinates retrieved from the CSD.

## 2. 2,2′:6′,2″-Terpyridine

The propensity for 2,2′:6′,2″-tpy to form chelates and thereby function as a monotopic ligand means that the introduction of a coordinatively non-innocent substituent into the 2,2′:6′,2″-tpy unit is typically required to transform an {M(2,2′:6′,2″-tpy)_2_} or {M(2,2′:6′,2″-tpy)X*_n_*} unit into a building block within an extended assembly. There are no examples in the CSD (v. 2020.3.1) [16] of coordination polymers or networks containing {Zn(2,2′:6′,2″-tpy)_2_} or {Cd(2,2′:6′,2″-tpy)_2_} units, and the assemblies described in the following sections incorporate {M(2,2′:6′,2″-tpy)X*_n_*} domains.

### 2.1. Assemblies with {M(4′-pyridinyl-2,2′:6′,2″-tpy)X_n_} (M = Zn, Cd) Building Blocks

**Figure 2:**
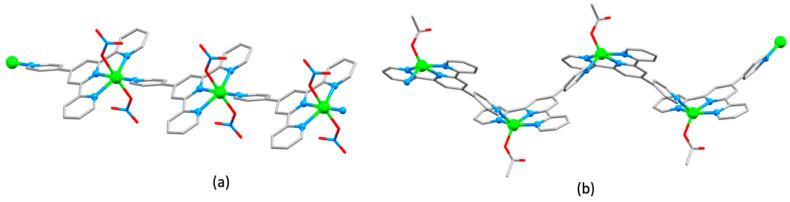
Parts of the 1D-coordination polymer chains in (**a**) [Zn(4′-(4-py)tpy)(NO_3_)_2_]*_n_* (CSD refcode UPUFOB) and (**b**) [{Zn(4′-(4-py)tpy)(OAc)}*_n_*]*^n^*^+^, isolated as the [PF_6_]^–^ salt (refcode WUJREA).

The incorporation of a 4′-pyridinyl substituent, in particular, 4′-pyridin-4-yl, into 2,2′:6′,2″-tpy is frequently employed to transform the ligand into a divergent metal-binding domain. The 1D-coordination polymer [Zn(4′-(4-py)tpy)(ONO_2_)_2_]*_n_* (Figure 2a) (4′-(4-py)tpy = 4′-pyridin-4-yl-2,2′:6′,2″-terpyridine) assembles under solvothermal conditions from Zn(NO_3_)_2_·6H_2_O and 4′-(4-py)tpy [18]. The chain is linear as a consequence of the octahedral Zn(II) centers with *trans*-nitrato ligands (Figure 2a). This contrasts with the zigzag cationic chain found in [{Zn(4′-(4-py)tpy)(OAc)}*_n_*][PF_6_]*_n_*, in which the Zn(II) centers are 5-coordinate (Figure 2b) [19]. 

**Figure 3:**
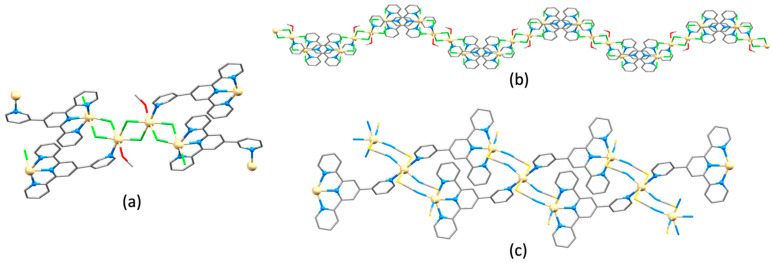
The structure of [Cd_2_Cl_4_(4′-(3-py)tpy)(MeOH)]*_n_* (CSD refcode RIXDOS): (**a**) ligands are connected by tetracadmium 4-connecting nodes, and (**b**) 2D-sheets assemble with a wave-like profile. (**c**) Part of the 1D-chain in [Cd_3_SCN)_6_(4′-(3-py)tpy)_2_]*_n_*·2*n*THF (refcode RIXDIM).

The vectorial properties of the M(4′-pyridinyl-2,2′:6′,2″-tpy) unit can be altered by the movement from a pyridin-4-yl to pyridin-3-yl or pyridin-2-yl substituent. Crystal growth by layering an MeOH solution of CdCl_2_·H_2_O over a THF solution of 4′-pyridin-3-yl-2,2′:6′,2″-tpy (4′-(3-py)tpy) under ambient conditions resulted in the assembly of [Cd_2_Cl_4_(4′-(3-py)tpy)(MeOH)]*_n_* in which the heterocyclic ligands are linked by tetranuclear 4-connecting nodes (Figure 3a). The structure propagates into a 2D-sheet with a wave-like profile (Figure 3b). In contrast, when thiocyanate replaces chloride ion, a 1D-chain assembles with ditopic ligands interconnected through trinuclear {Cd_3_(μ-NCS)_4_} units (Figure 3c). Face-to-face π-interactions between pairs of ligands arranged in a head-to-tail fashion are key packing interactions in both structures shown in Figure 3 [20]. Related assemblies incorporating [1,1′-biphenyl]-2,2′-dicarboxylate as a co-ligand have also been reported [21].

### 2.2. Assemblies with {Cd(4′-(4-(5-tetrazolato)phenyl)-2,2′:6′,2″-tpy)X_n_} Building Blocks

**Figure 4:**
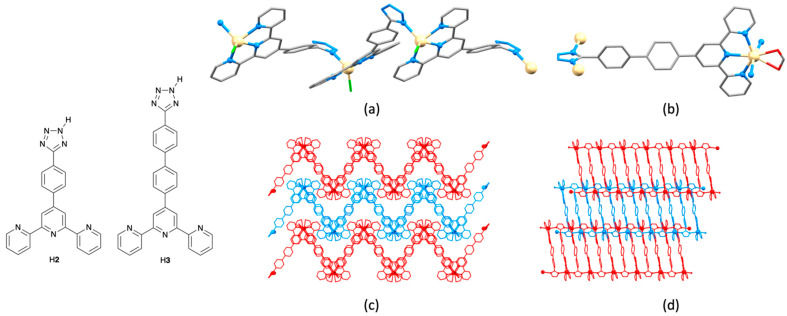
The structures of H**2** and H**3**, and of coordination polymers containing their conjugate bases. (**a**) Part of the 1D-chain in [CdCl(**2**)]*_n_*·*n*H_2_O (CSD refcode QIVRAR). (**b**) The building block in [Cd(O_2_CH)(**3**)]*_n_*·2*n*H_2_O (refcode PUCBUN). Packing of corrugated 2D-sheets in [Cd(O_2_CH)(**3**)]*_n_*·2*n*H_2_O viewed (**c**) down the *c*-axis and (**d**) down the *c*-axis, with adjacent layers colored red and blue.

Several cadmium(II)-containing coordination polymers featuring the conjugate bases of 4′-(4-(tetrazol-5-yl)phenyl)-2,2′:6′,2″-terpyridine, H**2**, and 4′-(4′-(tetrazol-5-yl)-[1,1′-biphenyl]-4-yl)-2,2′:6′,2″-terpyridine, H**3**, have been described, four as communications to the CSD [22,23,24,25]. [CdCl(**2**)]*_n_*·*n*H_2_O and [CdBr(**2**)]*_n_*·*n*H_2_O] are isostructural [22,23] and form 1D-chains (Figure 4a) with π-interactions between the phenylene ring of one ligand and one outer pyridine ring of the adjacent ligand (centroid…centroid = 3.84 Å). The corresponding iodido-derivative has a similar structure [24]. As Figure 4a shows, only one *N*-donor of the tetrazole is coordinated and ligand **2**^–^ is ditopic, with each Cd(II) center being 5-coordinate. In contrast, **3**^–^ acts as a tritopic ligand in [Cd(O_2_CH)(**3**)]*_n_*·2*n*H_2_O and the Cd(II) is 7-coordinate (the formate ion is bidentate, Figure 4b) [25,26]. The structure propagates into a corrugated 2D-sheet and the sheets pack together as shown in Figure 4c,d. Terpyridine H**3** was designed to possess intra-ligand charge-transfer (ICT) properties to facilitate singlet−triplet inter-system crossing. [Cd(O_2_CH)(**3**)]*_n_*·3*n*H_2_O [26] loses water while retaining the coordination network structure, and the process is fully reversible with a concomitant color change from yellow to orange. This remarkable material exhibits a long persistent luminescence in the dehydrated state, and this is suppressed when water re-enters the lattice. The incorporation of the triazole unit is key to these noteworthy photophysical properties, as is also demonstrated in [Cd(**2**)_2_]*_n_*·6.5*n*H_2_O, which exhibits a white-light emission when excited over a range of wavelengths (286−386 nm). The [Cd(**2**)_2_]*_n_* polymer consists of a double-stranded 1D chain with the Cd(II) center in a distorted tetrahedral environment; each 2,2′:6′,2″-tpy and each triazole unit coordinates to a Cd(II) center through only one *N*-donor [27].

### 2.3. Assemblies Containing Zn(II) and 2,2′:6′,2″-tpy Ligands Functionalized with Carboxylate Donors

**Scheme 5:**
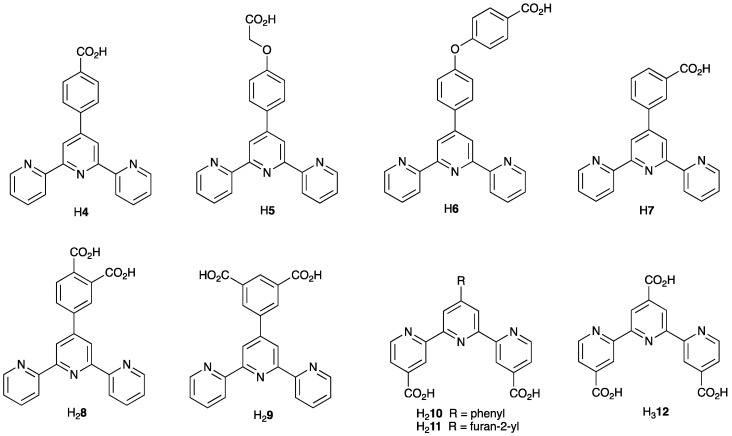
Carboxylic acid-functionalized 2,2′:6′,2″-tpy ligands.

**Figure 5:**
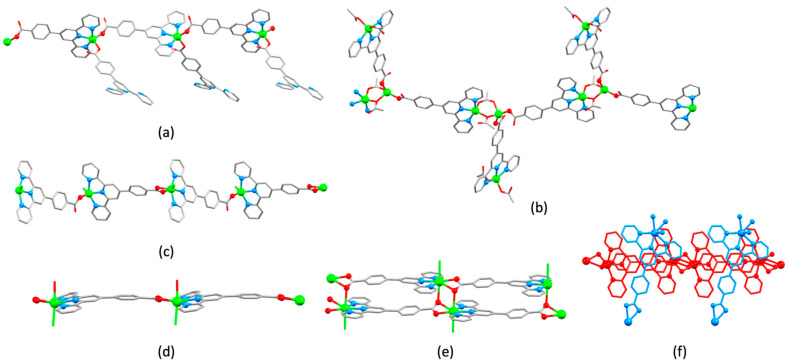
Parts of the 1D-coordination polymer chains in (**a**) [Zn(**4**)_2_]*_n_*·0.5*n*H_2_O (CSD refcode CEJBAW), (**b**) [{Zn_2_(μ-OAc)_2_}Zn(OAc)_2_(**4**)_2_]*_n_*·*n*H_2_O (recode DUBWUV), and (**c**) [Zn_2_Cl_2_(**4**)_2_]*_n_*·0.5*n*H_2_O (refcode GEYSOU). The structure of [ZnCl(**4**)]*_n_*·[ZnCl(OH_2_)(**4**)]*_n_* (refcode NUCBAR) contains both (**d**) single [ZnCl(OH_2_)(**4**)]*_n_* 1D-polymers, and (**e**) double-stranded [ZnCl(**4**)]*_n_* polymers; (**f**) π-stacking within the double-stranded chain (red) and between the two different chains (red and blue) is the dominant packing interaction in [ZnCl(**4**)]*_n_*·[ZnCl(OH_2_)(**4**)]*_n_*.

Scheme 5 presents the structures of 2,2′:6′,2″-tpy ligands bearing carboxylic acid functionalities that have been combined with zinc(II) or cadmium(II) in structurally characterized assemblies. In this section, we focus on zinc(II)-containing coordination polymers. Although the conjugate base of H**4** (Scheme 5) is regularly observed to interconnect Zn(II) centers into 1D-chains, the choice of zinc(II) salt and the reaction conditions are critical to the detailed assembly. Under hydrothermal conditions at pH 11.0, Zn(OAc)_2_·2H_2_O reacts with H**4** to give [Zn(**4**)_2_]*_n_*·0.5*n*H_2_O. The structure is a 1D-chain and contains 5-coordinate Zn(II) centers. As Figure 5a shows, there are two crystallographically independent ligands, one monodentate, binding only through the CO_2_^–^ unit and one utilizing both a chelating 2,2′:6′,2″-tpy and monodentate carboxylate to connect two Zn(II) centers [28]. The structure contrasts with the double-chain exhibited by [Cd(**4**)_2_]*_n_*·*n*H_2_O (see Section 2.4). Zinc(II) acetate reacted with H**4** under solvothermal conditions in DMF to produce [{Zn_2_(μ-OAc)_2_}Zn(OAc)_2_(**4**)_2_]*_n_*·*n*H_2_O (Figure 5b) [29]. The structure of the 1D-chain is related to that in [Zn(**4**)_2_]*_n_*·0.5*n*H_2_O in that only one of two crystallographically independent **4**^–^ ligands supports the backbone of the chain. However, the propensity for acetato ligands to be involved in the assembly of dinuclear zinc units makes the two chains in Figure 5a,b distinct from one another. Moreover, each pendant 2,2′:6′,2″-tpy domain in [{Zn_2_(μ-OAc)_2_}Zn(OAc)_2_(**4**)_2_]*_n_*·*n*H_2_O binds a Zn(OAc)_2_ unit (Figure 5b), in contrast to the non-coordinated 2,2′:6′,2″-tpy units in [Zn(**4**)_2_]*_n_*·0.5*n*H_2_O. Combining zinc(II) acetate with H**4** under solvothermal conditions (H_2_O/MeCN concentrated HCl, 160 °C) led to the assembly depicted in Figure 5c. Chlorido ligands are incorporated at the expense of acetato units and a simple 1D-chain forms in [Zn_2_Cl_2_(**4**)_2_]*_n_*·0.5*n*H_2_O. Although Figure 5c illustrates alternating 5- and 6-coordinate Zn(II) centers, the difference is described by the authors as ‘subtle′; the Zn–O distances are 2.047(4) and 2.468(5) Å for the bidentate CO_2_^–^, and 1.947(4) and 3.408(4) Å for the monodentate CO_2_^–^. An interesting feature of this structure is that adjacent chains interact through efficient face-to-face π-stacking of arene rings, and Hong and coworkers [30] describe the final assembly as a ‘2D + 2D→3D inclined polycatenated coordination polymer′ with the interchain π-stacking underpinning the higher dimensionality description. In the same work, Hong and coworkers showed that a change from zinc(II) acetate to nitrate and reaction with H**4** under the same conditions (solvothermal in H_2_O/MeCN with concentrated HCl at 160 °C) leads to the formation of [Zn(**4**)_2_]*_n_*·2.5*n*H_2_O. This comprises a 1D-chain analogous to that shown in Figure 5a. However, in [Zn(**4**)_2_]*_n_*·2.5*n*H_2_O (which crystallizes in the triclinic *P*–1 space group, refcode GEYTIP), the pendant 2,2′:6′,2″-tpy unit was presented with a *cis,cis* conformation [30] rather than the *trans,trans* conformation found in [Zn(**4**)_2_]*_n_*·0.5*n*H_2_O (space group *C*2*/c*) [28]. The latter is more usual for a non-coordinated 2,2′:6′,2″-tpy, and the atypical conformation in [Zn(**4**)_2_]*_n_*·2.5*n*H_2_O is not discussed by the authors. Finally in this series, a communication to the CSD [31] provides the structure of [ZnCl(**4**)]*_n_*·[ZnCl(OH_2_)(**4**)]*_n_*. Two different 1D-coordination polymers are present in the lattice. The first comprises single [ZnCl(OH_2_)(**4**)]*_n_* chains in which each 6-coordinate Zn(II) center is bound by chelating 2,2′:6′,2″-tpy, bidentate CO_2_^–^ and an aqua ligand. The backbone of the bridging ligand **4**^–^ is markedly bowed (Figure 5d). The second polymer [ZnCl(**4**)]*_n_* differs from the first in having no coordinated H_2_O and in possessing a double-stranded chain (Figure 5e). The Zn–O bond lengths within the carboxylate chelate ring are 2.226(5) and 2.399(3) Å, while the Zn–O distance between the two strands is 2.573(4) Å. Figure 5f illustrates the role of π-stacking within the double-stranded chain, and between the two different chains in [ZnCl(**4**)]*_n_*·[ZnCl(OH_2_)(**4**)]*_n_*. 

**Figure 6:**
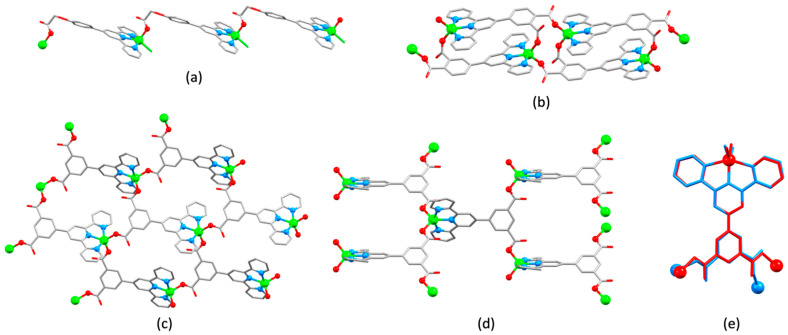
Parts of the 1D-coordination polymers in (**a**) [ZnCl(**5**)]*_n_*·0.5*n*H_2_O (CSD refcode FUJBET), and (**b**) [Zn(**8**)]*_n_* (refcode HOGYIO). (**c**) Part of the 2D-network in [Zn(**9**)]*_n_*·*n*H_2_O (CSD refcode KEGTAU) and (**d**) part of the 3D-net in [Zn(**9**)]*_n_*·2*n*MeOH·*n*NMP (CSD refcode KEGTEY, NMP = *N*-methyl-2-pyrrolidone). (**e**) An overlay of the building-block in each of [Zn(**9**)]*_n_*·*n*H_2_O (blue) and [Zn(**9**)]*_n_*·2*n*MeOH·*n*NMP (red) with Zn atoms in ball-representation showing the differences in the O–Zn vectors (bottom of the diagram).

Compounds H**5** and H**6** (see Section 2.4) are related to H**4** by the introduction of OCH_2_ and OC_6_H_4_ spacers, respectively (Scheme 5). The reaction of ZnCl_2_ with H**5** under hydrothermal conditions produced the 1D-coordination polymer [ZnCl(**5**)]*_n_*·0.5*n*H_2_O [32]. Structurally, this is similar to [Zn_2_Cl_2_(**4**)_2_]*_n_*·0.5*n*H_2_O (Figure 5c), but differs in having one crystallographically independent {ZnCl(tpy)} unit (Figure 6a). The lattice H_2_O molecules are involved in C–H…O hydrogen bonds and link the polymer chains into a 2D-sheet. Hu and coworkers extended this investigation to include the co-ligands terephthalic acid, fumaric acid, succinic acid and adipic acid [32]. Although coordination assemblies between Cd(II) and H**7** (Scheme 5) have been reported (see Section 2.4), no coordination polymers with Zn(II) have been structurally characterized. Both dicarboxylic acids H_2_**8** and H_2_**9** form extended structures with Zn(II). Under solvothermal conditions (DMF/H_2_O, 160 °C), ZnSO_4_·6H_2_O and H_2_**8** combine to give [Zn(**8**)]*_n_* (Figure 6b). The presence of the two CO_2_^–^ groups per ligand facilitates the assembly of a double-stranded chain [33]. This may be compared to the double-stranded chain found in [ZnCl(**4**)]*_n_*·[ZnCl(OH_2_)(**4**)]*_n_* (Figure 5e), but the additional carboxylate groups lead to distinct differences in the dinuclear building blocks in the 1D-chain. Polymer [Zn(**8**)]*_n_* was used as a sensor for Cr^3+^ ions through enhancement of fluorescence. H_2_**9** reacts with zinc(II) nitrate under solvothermal conditions (MeCN/H_2_O, 120 °C) to give single crystals of [Zn(**9**)]*_n_*·*n*H_2_O which possesses a 2D-network (Figure 6c). Each Zn(II) center is 5-coordinate and each **9**^2–^ ligand is tritopic through the use of a chelating tpy unit and two monodentate carboxylates. The assembly is, therefore, a {6^3^} net. When the reaction is carried out in *N*-methyl-2-pyrrolidone (NMP) and methanol at 95 °C, the assembly switches to a 3D-net (Figure 6d) with a *ths* topology. Although ligand **9**^2–^ again acts as a 3-connecting node in [Zn(**9**)]*_n_*·2*n*MeOH·*n*NMP; the difference in the [Zn(**9**)]*_n_* structure found for the two solvates arises from a change in directionality of the O–Zn vectors (Figure 6e). It is interesting to note that the change in topology is associated with a change from the monoclinic *P*2_1_/*n* space group to the orthorhombic *Fdd*2 space group. [Zn(**9**)]*_n_*·2*n*MeOH·*n*NMP exhibits a high capture capacity for CO_2_ with an unusual multistep gas-adsorption process [34].

**Figure 7:**
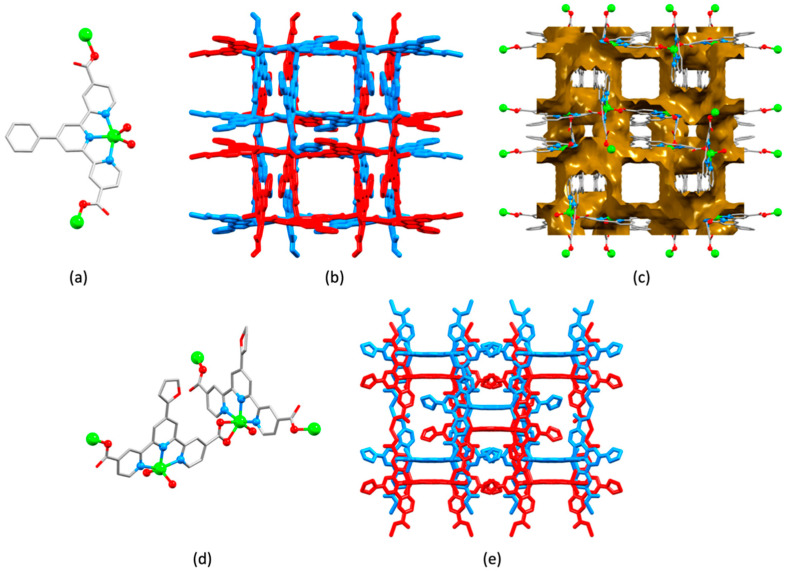
The structure of [Zn(**10**)]*_n_* (CSD refcode DECXOB): (**a**) the coordination zinc(II) environment, (**b**) interpenetrating *gis-c* 3D networks and (**c**) representation of the solvent accessible void-space (drawn using Mercury 2020.3.1 [17]). The structure of [Zn(**11**)]*_n_*·5*n*H_2_O (refcode PAFXUS): (**d**) the two independent zinc(II) and ligand environments, and (**e**) interpenetrating *sra* 3D nets viewed down the crystallographic *a*-axis.

Dicarboxylic acid H_2_**10** is representative of a series of compounds reported by Kruger and coworkers in which the R group (Scheme 5) is non-coordinating [35]. The reaction between Zn(NO_3_)_2_·6H_2_O and H_2_**10** under solvothermal conditions (DMF/H_2_O, concentrated HNO_3_, 90 °C) produced crystals of [Zn(**10**)]*_n_*. In this 3D-assembly, the local environment at each Zn center (Figure 7a) is the same as in [Zn(**8**)]*_n_* and [Zn(**9**)]*_n_* (Figure 6b–d). Kruger describes the unit shown in Figure 7a as a 4-connecting node, and the assembly (as well as those with analogous ligands with R = 2-methylphenyl, 4-chlorophenyl, 4-nitrophenyl, thien-2-yl, thien-3-yl, pyridin-4-yl) propagates into a zeolitic gismondine *gis-c* (4^3^·6^2^·8) topology; the framework is 2-fold interpenetrating (Figure 7b). Channels with a square cross-section (ca. 9.0 Å across) follow the *c*-axis and Figure 7c illustrates the solvent accessible void space. For [Zn(**10**)]*_n_*, this is 40%, and varies from 32% to 41% for the family of structures. Gas adsorption measurements using N_2_, H_2_, CO_2_ and CH_4_ confirm that the solid-state structures exhibit permanent porosity [35]. The reaction of dicarboxylic acid H_2_**11** (Scheme 5) with zinc(II) acetate has been investigated by Lu and coworkers. Despite the structural similarity between H_2_**11** and H_2_**10**, the 3D-assembly observed for [Zn(**11**)]*_n_*in the pentahydrate contrasts with that found for [Zn(**10**)]*_n_* and its analogs (see above). [Zn(**11**)]*_n_*·5*n*H_2_O was formed under hydrothermal conditions starting with K_2_**11**; the pH was adjusted to 2.0 using MeCO_2_H [36]. These conditions contrast with those employed in Kruger′s work [35]. There are two independent Zn centers and ligands in the solid-state structure of [Zn(**11**)]*_n_*·5*n*H_2_O, as shown in Figure 7d. The assembly extends into a 4-connected *sra* net with (4^2^.6^3^.8) topology, and 2-fold interpenetration occurs (Figure 7e). The structure possesses interconnected channels that run parallel to the *a*, *b* and *c*-axes, although, as Figure 7e illustrates, the furan-2-yl substituents are accommodated within some of the channels. The solvent-accessible void space is ca. 29% [36]. The same *sra* topology was reported by Wang, Sun and coworkers for [Zn_2_(**11**)_2_]*_n_*·5*n*H_2_O (CSD refcode TACTUP) which was prepared solvothermally from Zn(NO_3_)_2_·6H_2_O and H_2_**11** [37]. A comparison of the two structure-types depicted in Figure 7 serves to emphasize the variation that is possible with similar building blocks. 

**Figure 8:**
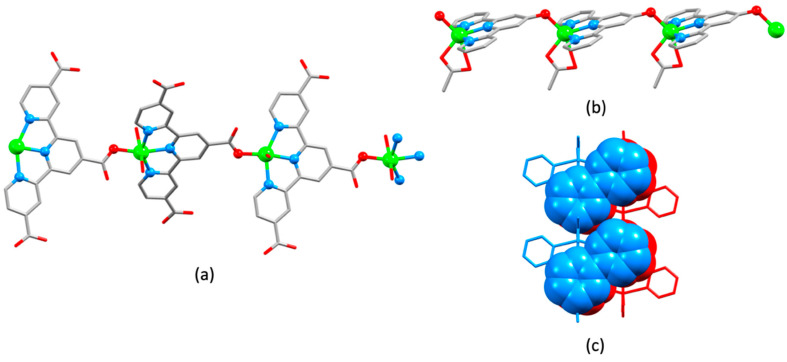
Part of the 1D-coordination polymers in (**a**) [Zn(H**12**)(OH_2_)_2_]*_n_*·2*n*H_2_O (CSD refcode CESRAW), and (**b**) [Zn(4′-O-2,2′:6′,2″-tpy)(OAc)]*_n_* (refcode WUKBAH). (**c**) Adjacent chains in [Zn(4′-O-2,2′:6′,2″-tpy)(OAc)]*_n_* associate through face-to-face π-stacking.

The introduction of a third carboxylic acid functionality in H_3_**12** (Scheme 5) might be expected to lead to a high-dimensionality assembly. However, the hydrothermal reaction of H_3_**12** with zinc(II) sulfate led to a 1D-coordination polymer [Zn(H**12**)(OH_2_)_2_]*_n_*·2*n*H_2_O in which only one CO_2_^–^ group is involved in coordination. Although written here as H**12**^2–^, the protonation state of the coordinated ligand is unclear. Each Zn center is 6-coordinate (Figure 8a), and the chain is built up by a glide plane. The coordination polymer functions as a sensor for Hg^2+^ in aqueous solutions with its fluorescence being quenched by trace amounts of the heavy metal ion [38].

### 2.4. An Assembly Containing Zn(II) and the Conjugate Base of 4′-Hydroxy-2,2′:6′,2″-terpyridine

The compound [Zn(4′-O-2,2′:6′,2″-tpy)(OAc)]*_n_* is an unusual example of a coordination polymer employing the conjugate base of 4′-hydroxy-2,2′:6′,2″-terpyridine (4′-HO-2,2′:6′,2″-tpy). This appears to be the only structurally characterized coordination polymer of any metal ion incorporating this ligand. The zinc(II) complex was prepared on a preparative scale from the reaction of Zn(OAc)_2_·2H_2_O with 4′-HO-2,2′:6′,2″-tpy, and single crystals grown by diffusion of *n*-hexane into a methanol solution of the compound proved to be [Zn(4′-O-2,2′:6′,2″-tpy)(OAc)]*_n_*. NMR spectroscopic data for the bulk material are consistent with a degree of dissociation in solution. The structure of the 1D-polymer is shown in Figure 8b. The Zn–O_tpy_ bond length of 1.999(4) Å compares with distances of 2.028(4) and 2.576(4) Å for the coordinated acetato ligand. Neighboring chains are associated through π-stacking of 2,2′:6′,2″-tpy domains (Figure 8c) [19].

### 2.5. Assemblies Containing Cd(II) and 2,2′:6′,2″-tpy Ligands Functionalized with Carboxylate or Sulfonate Donors

**Figure 9:**
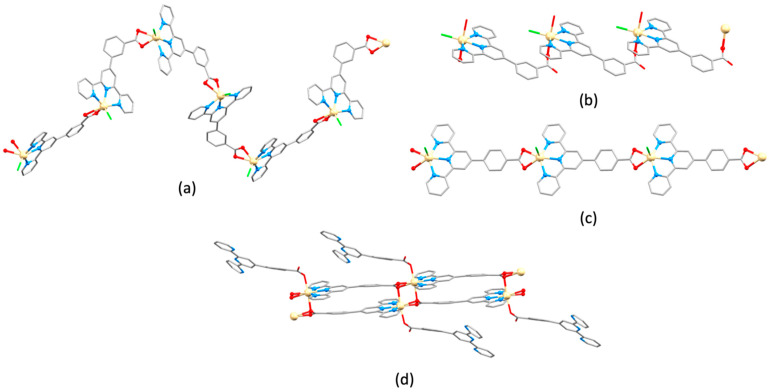
Part of a 1D-chain in (**a**) [Cd_2_(**7**)_2_Cl_2_]*_n_*·*n*H_2_O (recode OBOFET), (**b**) [Cd(**7**)Cl(H_2_O)]*_n_·n*[Cd(H**7**)Cl_2_]·*n*H_2_O (refcode OBIFIX), (**c**) [Cd(**4**)Cl(H_2_O)]*_n_·*3.75*n*H_2_O (refcode ZUDPAR), and (**d**) [Cd(**4**)_2_]*_n_*·*n*H_2_O (refcode KABTOY).

The conjugate base of H**7** (Scheme 5) forms 1D-coordination polymers when reacted with CdCl_2_·2.5H_2_O under solvothermal conditions. Depending upon the pH, the isolated single crystals were determined to be [Cd_2_(**7**)_2_Cl_2_]*_n_*·*n*H_2_O or [Cd(**7**)Cl(H_2_O)]*_n_·n*[Cd(H**7**)Cl_2_]·*n*H_2_O. Figure 9a illustrates the polymer chain in the former complex; the 6-coordinate Cd(II) center is bound by chelating CO_2_^–^ and 2,2′:6′,2″-tpy domains and a chlorido ligand. The co-crystal resulting from assembly under acidic conditions contains 1D [Cd(**7**)Cl(H_2_O)]*_n_* chains with octahedral Cd(II) (Figure 9b) and discrete [Cd(H**7**)Cl_2_] molecules with 5-coordinate Cd(II) centers [39]. On going from H**7** to H**4** (Scheme 5), the vectorial properties of the ligand change, and the effect on the structure of the 1D chain in [Cd(**4**)Cl(H_2_O)]*_n_·*3.75*n*H_2_O [40] compared to that in [Cd_2_(**7**)_2_Cl_2_]*_n_*·*n*H_2_O can be seen by comparing Figure 9b,c. Tseng et al. [40] also illustrated that introducing a co-ligand (thiophene-2,5-dicarboxylic acid) had a significant impact on the assembly, directing it towards an 8-fold (4,2) interpenetrated network. The reaction of H**4** with cadmium(II) nitrate under hydrothermal conditions at pH 7.5 resulted in the formation of single crystals of [Cd(**4**)_2_]*_n_*·*n*H_2_O. The assembly consists of a double chain in which half of the ligands bridge between Cd(II) centers and half coordinate only through the carboxylate group (Figure 9d). The head-to-tail arrangement of the ligands along the chain leads to efficient inter-ligand π-stacking [41]. A similar situation is observed in the 1D-coordination polymer [Cd(**6**)_2_]*_n_*·*n*H_2_O (see Scheme 5 for the structure of H**6**) in which half of the ligands exhibit a pendant, non-coordinated 2,2′:6′,2″-tpy domain. The 1D-chain comprises Cd(II) centers linked by ditopic ligands **6**^–^, and each Cd(II) is additionally bound by a monodentate **6**^–^ ligand. Once again, π-stacking between 2,2′:6′,2″-tpy units is a dominant packing interaction [42].

**Figure 10:**
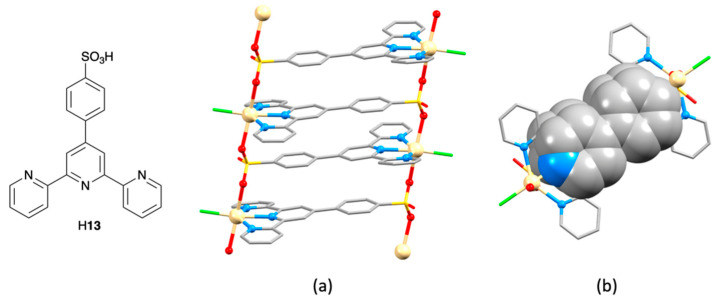
The structure of H**13.** (**a**) Part of the 1D-ladder in [Cd(**13**)Cl]*_n_* (CSD refcode ZIQGUD) and (**b**) space-filling representation of the phenylpyridine units which are stacked in a head-to-tail arrangement down the ladder.

Replacing the carboxylic acid functionality in ligand H**4** by a sulfonic acid to give H**13** (Figure 6) has an interesting effect on the assembly. The reaction between Na**13** and CdCl_2_ under hydrothermal conditions produced [Cd(**13**)Cl]*_n_* which possesses a 1D-ladder structure. The compound was prepared as a model system with which to compare the photophysical behaviors of lanthanoid(III) metal analogs, and the structure is described only in the supporting information in the original work [43]. Figure 10a shows the ladder assembly, in which the rungs comprise the heterocyclic ligands, and the struts comprise the Cd(II) centers and sulfonate groups. The phenylpyridine units of the ligands are arranged in a head-to-tail fashion and engage in face-to-face π-stacking interactions (Figure 10b).

## 3. 4,2′:6′,4″-Terpyridine

### 3.1. A 1D-Coordination Polymer with an Unfunctionalized 4,2′:6′,4″-tpy

**Figure 11:**
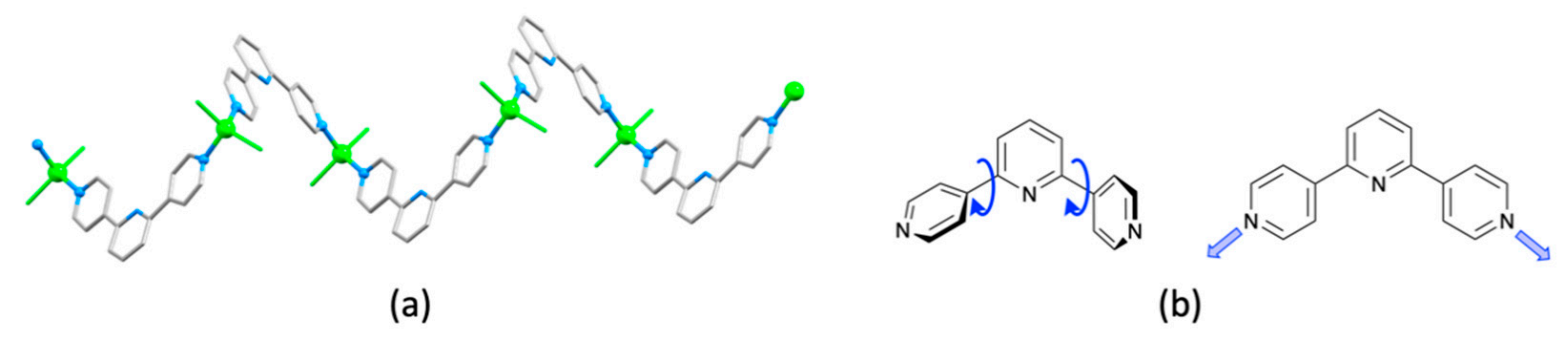
(**a**) Part of the 1D-chain in [Zn(4,2′:6′,4″-tpy)Cl_2_]*_n_* (CSD refcode GAQYUS). (**b**) Rotation about the inter-ring C–C bonds does not change the vectorial properties of the ditopic 4,2′:6′,4″-tpy metal-binding domain.

In 1998, González-Garmendia and coworkers reported the synthesis and single crystal structure of [Zn(4,2′:6′,4″-tpy)Cl_2_]*_n_* (Figure 11a), which was the first example of a coordination polymer containing the 4,2′:6′,4″-tpy metal-binding domain [44]. The structure demonstrates several features that recur in the related structures, described below. Most significantly, the central pyridine ring 4,2′:6′,4″-tpy is non-coordinating. At the time of writing this review, this feature was common to all structurally characterized coordination compounds involving 4,2′:6′,4″-tpy and its derivatives. Secondly, the V-shaped building block of 4,2′:6′,4″-tpy is moderately rigid, with the directional properties of the outer *N*-donors being independent of conformational changes involving rotation about the inter-ring C–C bonds (Figure 11b). Compare this with the situation for 3,2′:6′,3″-tpy (Scheme 4). [Zn(4,2′:6′,4″-tpy)Cl_2_]*_n_* crystallizes as a heterochiral polymer with equal numbers of left-handed (*M*) and right-handed (*P*) helices in the crystal lattice. Such helical 1D-polymers feature regularly in the structures of Zn(II)/4,2′:6′,4″-tpy assemblies, although the pitch of the helix is markedly varied [45]. However, not all [Zn(4′-X-4,2′:6′,4″-tpy)Y_2_]*_n_* (X is a coordinatively innocent group and Y^–^ is a monodentate ligand) are helical (see below). The Zn…Zn distance between adjacent Zn atoms in a chain in [Zn(4,2′:6′,4″-tpy)Cl_2_]*_n_* is 12.528(4) Å (Table 1).

### 3.2. Assemblies Containing Zinc(II) and 4′-Functionalized 4,2′:6′,4″-tpy Ligands with Coordinatively Innocent Substituents

**Scheme 6:**
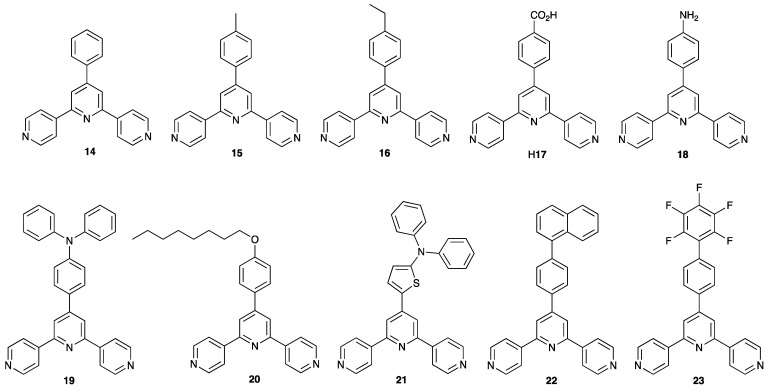
Structures of 4′-functionalized 4,2′:6′,4″-tpy ligands which form [Zn(4′-X-4,2′:6′,4″-tpy)Cl_2_]*_n_* 1D-coordination polymers.

**Figure 12:**
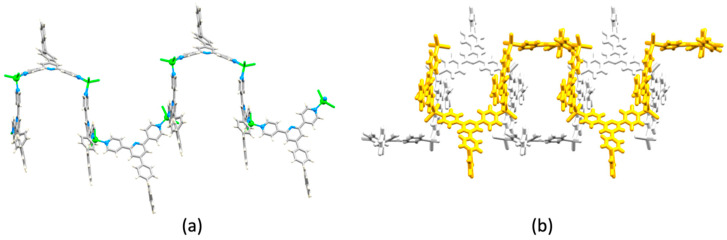
(**a**) Part of one chain of [Zn(**22**)Cl_2_]*_n_* (CSD refcode QOJBOI) showing the crenelated profile. (**b**) Adjacent chains in [Zn(**23**)Cl_2_]*_n_* are related by inversion (refcode QOJBAU).

**Figure 13:**
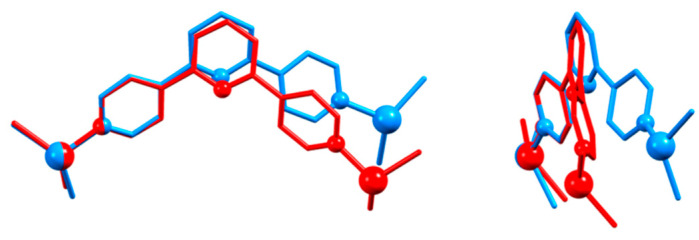
Two views of overlays of {(ZnCl_2_)_2_(4,2′:6′,4″-tpy)} units excised from the coordination polymer chains in [Zn(**22**)Cl_2_]*_n_* (red, Zn…Zn = 12.325(2) Å) and [Zn(**23**)Cl_2_]*_n_* (blue, Zn…Zn = 13.062(1) Å). (CSD refcodes QOJBOI and QOJBAU, see Table 1).

A wide variety of coordinatively innocent 4′-functionalities has been introduced into 4,2′:6′,4″-tpy ligands; note that 4′-(pyridinyl)-substituted ligands and their zinc(II) complexes are discussed later in Section 3.5. Scheme 6 illustrates those ligands (**14–16**, H**17**, **18–23**) which react with ZnCl_2_ to produce 1D-coordination polymers. Some details of the structures are given in Table 1, and can be compared with [Zn(4,2′:6′,4″-tpy)Cl_2_]*_n_* (Section 3.1). The chains are typically built up along a 2-fold screw axis (refcodes GAQYUS [44], FEPRUO [46], LOCTED [47], NOGFOF [47], DOMBIS [49], AJURIG [51], ELAMOV [14]), a 2-fold rotation axis (NOGFIZ [47], DIRMEY [48]), or a glide plane (SEBRID [50]). The coordination polymers [Zn(**22**)Cl_2_]*_n_* and [Zn(**23**)Cl_2_]*_n_* with 4′-(4-(1-naphthyl)phenyl)- and 4′-(2′,3′,4′,5′,6′-pentafluorobiphenyl-4-yl)-substituents, respectively, are of particular note. The asymmetric unit in each possesses four crystallographically independent Zn atoms, and the chain is built up by translational symmetry to generate a polymer with a distinctive crenelated profile (Figure 12a). Adjacent chains are related to each other by inversion (Figure 12b). Each embrasure in the crenelation can be likened to three sides of a metallocycle and it is significant that the assembly of [Zn(**23**)Cl_2_]*_n_* polymer chains competes with the formation of the metallosquare [Zn(**23**)Cl_2_]_4_ [52]. In Figure 11b, we described the V-shaped 4,2′:6′,4″-tpy as a moderately rigid building block. In Table 1, the range of Zn…Zn distances between adjacent zinc atoms in a chain (12.325(2)–13.062(1) Å) gives an indication of the flexibility of 4,2′:6′,4″-tpy, and Figure 13 illustrates that the ligand is noticeably bowed when associated with shorter Zn…Zn distances. We have previously commented upon the bowing of terpyridine backbones in solid-state structures [53]; this mode of flexibility is additional to conformational changes arising from bond rotation.

**Figure 14:**
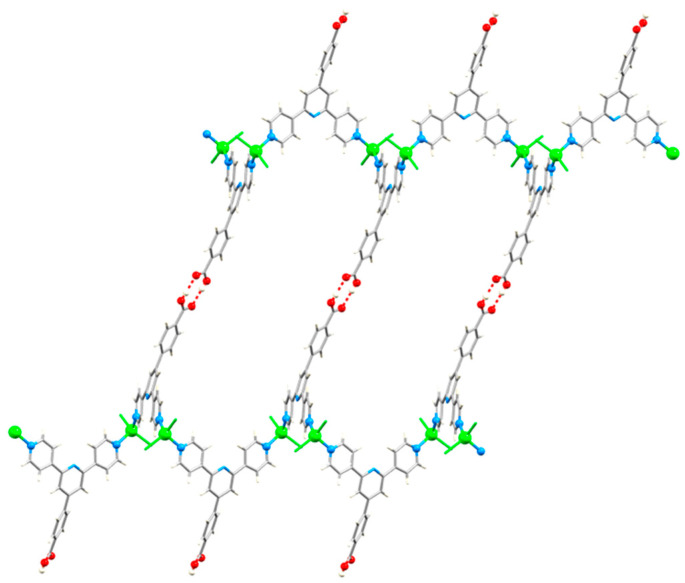
Hydrogen-bonded polymer chains in [Zn_2_(H**17**)_2_Cl_4_]*_n_*·2*n*EtOH·*n*H_2_O associate into a 2D-sheet (CSD refcode DIRMEY).

Ligand H**17** (Scheme 6) is noteworthy because it remains protonated in [Zn_2_(H**17**)_2_Cl_4_]*_n_*·2*n*EtOH·*n*H_2_O, in contrast to the assemblies containing carboxylates described in Section 3.4. Pairs of peripheral CO_2_H groups in adjacent chains engage in hydrogen-bonding, leading to interconnection of the chiral [Zn_2_(H**17**)_2_Cl_4_]*_n_* chains into 2D-sheets (Figure 14). Each layer contains alternating left- and right-handed helical polymer chains [48].

**Scheme 7:**
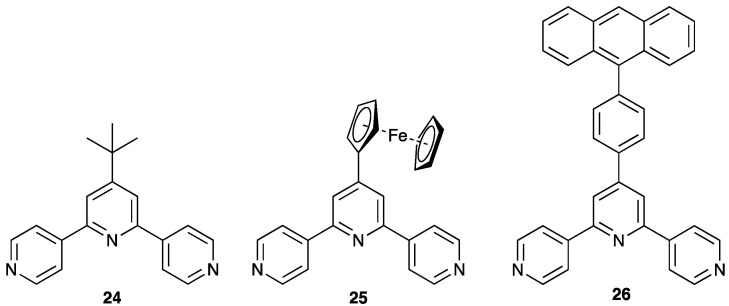
Structures of 4′-functionalized 4,2′:6′,4″-tpy ligands **24** and **25** without organic spacers in the 4′-position, and the structure of **26**.

A number of 1D-coordination polymers related to those described above incorporate different ZnX_2_ units (X^–^ = I^–^, NCS^–^, monodentate AcO^–^) and these are summarized in Table 2. The structures of ligands **24** and **25** are depicted in Scheme 7. Each of the [Zn(**25**)(NCS)_2_]*_n_*and [Zn(**25**)(OAc)_2_]*_n_* chains (TUNNIB and TUNCIQ in Table 2) is built up by a glide plane and is achiral, whereas each chain in [Zn(**14**)(OAc)_2_]*_n_*, [Zn(**21**)I_2_]*_n_*, [Zn(**24**)I_2_]*_n_*, and [Zn(**25**)I_2_]*_n_* (CUXDOP, ELAMEL, FAKRUG and TUNKEU in Table 2) is propagated along a 2-fold screw axis, with the lattice containing equal numbers of right- and left-handed helical polymers [14,54,55,56]. A second solvate of [Zn(**25**)(OAc)_2_]*_n_* has been structurally characterized (NEFWED in Table 2), and although the chains in [Zn(**25**)(OAc)_2_]*_n_*·2*n*H_2_O [56] and [Zn(**25**)(OAc)_2_]*_n_*·*n*MeOH·*n*H_2_O [57] are very similar, the change in space group (Table 2) leads to the 1D-polymer in [Zn(**25**)(OAc)_2_]*_n_*·*n*MeOH·*n*H_2_O being built up by translational symmetry. Crystals of [Zn(**14**)(OAc)_2_]*_n_* were formed under ambient conditions (by layering) after a period of about a month. In contrast, crystals harvested from the same crystallization tube after three days comprised the 1D-polymer [Zn_2_(OAc)_4_(**14**)]*_n_* with {Zn_2_(OAc)_4_} paddle-wheel building blocks [54] (see Section 3.3). Both [Zn_2_(OAc)_4_(**14**)]*_n_* and [Zn(**14**)(OAc)_2_]*_n_* are emissive, and exposure to CF_3_CO_2_H vapor resulted in a red-shift in the emission from 384 to 435 nm for [Zn_2_(OAc)_4_(**14**)]*_n_*, and from 390 to 441 nm for [Zn(**14**)(OAc)_2_]*_n_*. This most likely arises from protonation of the central pyridine ring of coordinated ligand **14**. However, attempts to alkylate the polymers using MeI or EtI resulted in degradation of the chain and formation of [R_2_**14**][ZnI_4_], ROAc and Zn(OAc)_2_ (R = Me, Et) [54]. Helical polymers are also formed in reactions of zinc(II) acetate with 4′-(4-(anthracen-9-yl)phenyl)-4,2′:6′,4″-terpyridine (**26**), and both homochiral polymers and racemates were isolated from the same crystallization tubes (Table 2) [58].

Earlier, we detailed how the assembly of the polymeric [Zn(**23**)Cl_2_]*_n_* competes with the formation of the metallosquare [Zn(**23**)Cl_2_]_4_ [52]. In this case, both polymer and discrete metallomacrocycle were observed in the same crystallization tube. In other cases, only cyclic or polymeric structures were isolated and structurally characterized. Wu, Tian and coworkers reported that reactions of **25** (Scheme 7) with ZnCl_2_ and ZnBr_2_ under conditions of crystal growth by layering (MeOH solution of ZnX_2_ layered over a CHCl_3_ solution of **25**) gave single crystals of the metallohexacycles [Zn_6_(**25**)_6_Cl_12_]·6H_2_O·6CHCl_3_ and [Zn_6_(**25**)_6_Br_12_]·6H_2_O·6CHCl_3_ [56]. In contrast, we have observed that, under similar conditions of crystal growth to those used by Wu and Tian [56], ZnCl_2_ and ligand **25** react to give the metallosquare [Zn_4_(**25**)_4_Cl_8_]·2CHCl_3_ [57]. The factors that direct the assembly are not understood [59], and, in the absence of powder X-ray diffraction (PXRD) data for the bulk material, it is difficult to assess the competitive nature of these assemblies. We return to the critical need for routine PXRD later.

### 3.3. Assemblies Containing 4′-Functionalized 4,2′:6′,4″-tpy Ligands Connected by {Zn_2_μ-OAc)_4_} Paddle-Wheel Units

**Scheme 8:**
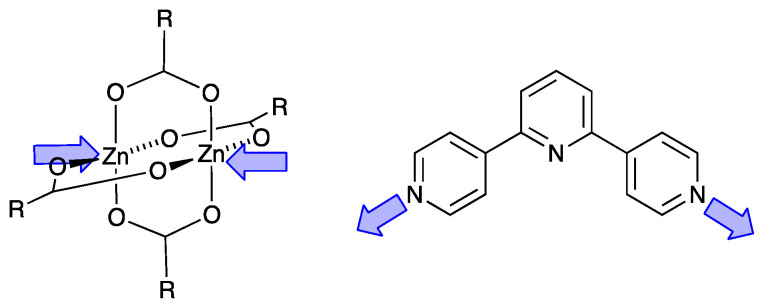
Linear and V-shaped directional properties, respectively, of the Lewis acidic {Zn_2_(μ-OAc)_4_} paddle-wheel and Lewis basic 4,2′:6′,4″-tpy ligand building blocks.

**Scheme 9:**
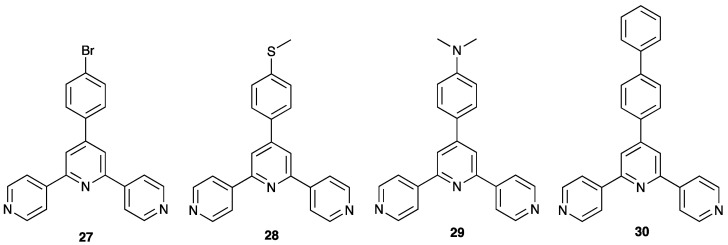
Ligands that form [Zn_2_(μ-OAc)_4_(4′-X-4,2′:6′,4″-tpy)]*_n_* type 1D-zigzag polymers. See Scheme 6 for ligand **14**.

**Figure 15:**
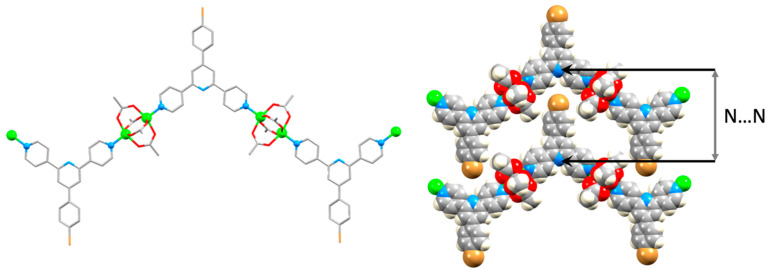
Part of the 1D-zigzag coordination polymer chain in [Zn_2_(μ-OAc)_4_(4′-(4-BrC_6_H_4_)-4,2′:6′,4″-tpy)]*_n_* (CSD refcode CUXCOO) and a space-filling representation of parts of two adjacent chains showing packing to form 2D-layers. The defined N…N distance gives a measure of the separation between chains in a given layer (see text).

Although Table 2 shows examples of 1D-coordination polymers with mononuclear {Zn(OAc)_2_} building blocks, the tendency for dimerization to form {Zn_2_(μ-OAc)_4_} paddle-wheel units means that reactions between zinc(II) acetate and 4′-functionalized 4,2′:6′,4″-tpy ligand might typically be expected to lead to 1D-chains of general formula [Zn_2_(μ-OAc)_4_L]*_n_*. The combination of the linear and V-shaped directional properties, respectively, of the paddle-wheel and the ligand (Scheme 8) results in the formation of zigzag chains. A characteristic example is [Zn_2_(μ-OAc)_4_(**27**)]*_n_* (see Scheme 9 for **27** and related ligands), the structure of which is shown in Figure 15 [60]. This also shows the typical packing of adjacent zigzag chains into 2D-sheets. Packing of the layers involves face-to-face π-stacking of 4,2′:6′,4″-tpy domains, with the acetato groups from one layer accommodated in pockets in an adjacent layer. Structures analogous to that observed for [Zn_2_(μ-OAc)_4_(**27**)]*_n_* are found for [Zn_2_(μ-OAc)_4_(**14**)]*_n_*·0.3*n*CH_2_Cl_2_ (refcode CUXDUV) [54], [Zn_2_(μ-OAc)_4_(**28**)]*_n_*(refcode CUXCUU) [60], and [Zn_2_(μ-OAc)_4_(**29**)]*_n_*(refcode QOTROI) [61]. Note that in the case of [Zn_2_(μ-OAc)_4_(**14**)]*_n_*·0.3*n*CH_2_Cl_2_, crystals grew from a layering experiment under ambient conditions within 3 days and the polymer was isolated in 82% yield. However, over longer crystallization periods under the same conditions, the colorless needles of [Zn_2_(μ-OAc)_4_(**14**)]*_n_*·0.3*n*CH_2_Cl_2_ which initially formed were largely replaced by pale-yellow blocks of [Zn(**14**)(OAc)_2_]*_n_* (see Table 2) [54]. Once again, this highlights the competitive nature of the assembly processes, and underlines the need for routine analysis of bulk samples by using PXRD.

**Figure 16:**
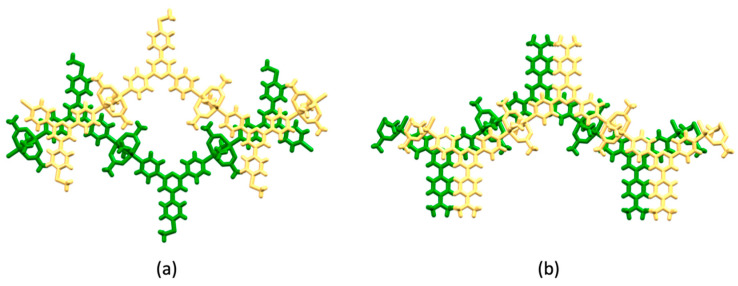
In [Zn_2_(μ-OAc)_4_(4′-X-4,2′:6′,4″-tpy)]*_n_* type 1D-zigzag polymers, face-to-face π-stacking occurs between chains, and the arrangement is influenced by the X substituent. For example: (**a**) [Zn_2_(μ-OAc)_4_(**28**)]*_n_* (CSD refcode CUXCUU), and (**b**) [Zn_2_(μ-OAc)_4_(**29**)]*_n_* (refcode QOTROI). Note that the mode of packing into 2D-sheets is analogous in both compounds (see Figure 15).

A zigzag polymer is also assembled when ligand **30** reacts with zinc(II) acetate under conditions of crystal growth by layering. In [Zn_2_(μ-OAc)_4_(**30**)]*_n_*, (CSD refcode RIJFUN), face-to-face π-stacking of both pairs of biphenyl domains and pairs of 4,2′:6′,4″-tpy units contribute to packing interactions [62]. The zigzag chains are arranged into 2D-sheets in a similar manner to the chains in polymers containing **14**, **27**, **28**, and **29**, but the greater steric demands of the biphenyl unit force the chains further apart. The N…N distance defined in Figure 15 is 16.151 Å in [Zn_2_(μ-OAc)_4_(**30**)]*_n_*, and this compares to 15.294, 15.077, 14.902 and 14.898 Å in the assemblies with **14**, **27**, **28** and **29**. While the mode of packing of chains into 2D-sheets remains the same in [Zn_2_(μ-OAc)_4_(4′-X-4,2′:6′,4″-tpy)]*_n_* assemblies with 4′-X-4,2′:6′,4″-tpy = **14**, **27**, **28**, **29**, and **30**, the different substituents influence the arrangement of the layers with respect to one another (as exemplified in Figure 16) in order to optimize packing interactions. Note that the solvent-accessible void space is small, and within this series, only [Zn_2_(μ-OAc)_4_(**14**)]*_n_*·0.3*n*CH_2_Cl_2_ with the 4′-phenyl substituent in ligand **14** incorporates solvent.

**Figure 17:**
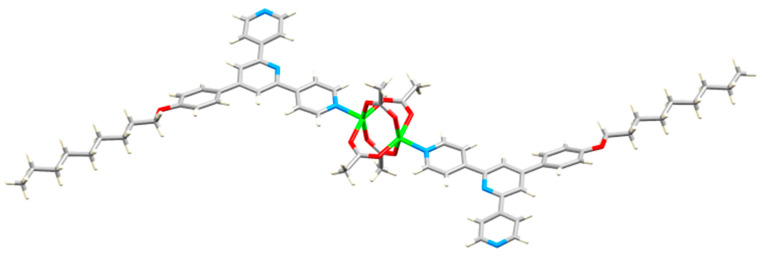
The structure of the discrete complex [Zn_2_(μ-OAc)_4_(4′-(4-(*^n^*nonyloxy)phenyl)-4,2′:6′,4″-tpy)_2_] (CSD refcode SOXVAE) in which the ligands are monodentate.

Systematic investigations are typically lacking for coordination polymers containing a given family of ligands. We have carried out one such study for combinations of zinc(II) acetate and 4′-(4-ROC_6_H_4_)-4,2′:6′,4″-tpy ligands in which R = methyl, ethyl, *^n^*propyl, *^n^*butyl, *^n^*pentyl, *^n^*hexyl, *^n^*heptyl, *^n^*octyl, *^n^*nonyl and *^n^*decyl [63]. For the smaller substituents (OMe, OEt, O*^n^*Pr), [Zn_2_(μ-OAc)_4_(4′-X-4,2′:6′,4″-tpy)]*_n_* assemblies were observed (CSD refcodes SOXSEF, SOXSIJ, SOXSOP) which are similar to that found for [Zn_2_(μ-OAc)_4_(**28**)]*_n_* with the SMe substituent (CSD refcode CUXCUU) [60]. The N…N distance (defined in Figure 15) increases from 15.214 to 16.077 Å from OMe to O*^n^*Pr. On going to the longer chains, several changes occur. Firstly, there is competition between the assembly of a coordination polymer and a discrete complex of type [Zn_2_(μ-OAc)_4_(4′-(4-ROC_6_H_4_)-4,2′:6′,4″-tpy)_2_]. The latter is the sole isolated product for the ligands with *^n^*octyl, *^n^*nonyl and *^n^*decyl tails, and the structure of one example is shown in Figure 17. Single-crystal structures for the coordination polymers with the *^n^*pentyl, *^n^*hexyl and *^n^*heptyl tails (refcodes SOXTUW, SOXGIX and SOXTAC, respectively) confirm the presence of 1D-zigzig polymer chains and packing into 2D sheets in the manner depicted in Figure 15; cavities within a sheet are occupied by solvent. Changes in the packing of sheets demonstrate the response to the increased steric demands of the alkyloxy tails [63].

**Figure 18:**
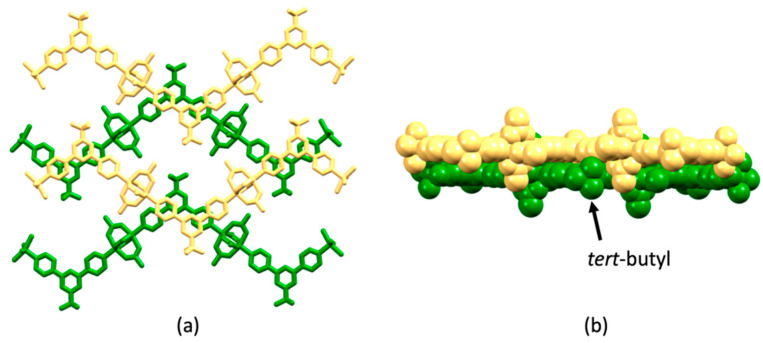
(**a**) Stacking of zigzag chains in [Zn_2_(μ-OAc)_4_(**24**)]*_n_* (CSD refcode FAKROA) with π-stacking between 4,2′:6′,4″-tpy units, and (**b**) a space-filling representation of the chains shown in (**a**) viewed from the side to show the location of a *tert*-butyl group between a pair of acetato units in the adjacent layer.

In all the assemblies discussed in the section so far, the ligands have contained a phenyl group or phenylene spacer in the 4′-position of the 4,2′:6′,4″-tpy. The structural similarities between the ligands in [Zn_2_(μ-OAc)_4_(4′-X-4,2′:6′,4″-tpy)]*_n_* 1D-coordination polymers result in similar packing interactions within this series of compounds. In contrast, ligand **24** (Scheme 7) contains a *tert*-butyl substituent attached directly to the central ring of the 4,2′:6′,4″-tpy metal-binding domain. Once again, zigzig chains that pack together to form 2D-sheets are assembled, and it is significant that in [Zn_2_(μ-OAc)_4_(**24**)]*_n_*, packing between the zigzag 1D-chains, is dominated by face-to-face π-stacking of 4,2′:6′,4″-tpy units (Figure 18a). The ball-like *tert*-butyl groups are accommodated in cavities between acetato units in the next sheet (Figure 18b) [55]. Note how the stacking of chains in [Zn_2_(μ-OAc)_4_(**24**)]*_n_* differs from the arrangement in chains with phenylene spacers (compare Figure 18a with Figure 16). The N…N separation in [Zn_2_(μ-OAc)_4_(**24**)]*_n_* (see Figure 15) is 14.892 Å.

**Figure 19:**
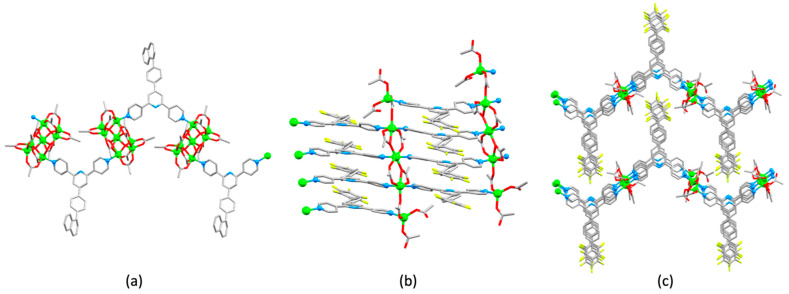
(**a**) Part of one helical chain in [2{Zn_7_(μ-OAc)_10_(μ_4_-O)_2_(**22**)}]*_n_*·*n*CH_2_Cl_2_ (CSD refcode OFIWAD). (**b**) Part of the 1D, quadruple-stranded chain in [Zn_5_(OAc)_10_(**23**)_4_]*_n_*·11*n*H_2_O (refcode QIJHEY) and (**c**) packing of adjacent chains to form a thick 2D-sheet (compare with Figure 15).

Before leaving this section, two examples of reactions of zinc(II) acetate with 4,2′:6′,4″-tpy ligands illustrate, not for the first time, that serendipity contributes significantly to assembly processes. Ligand **22** (Scheme 6) and Zn(OAc)_2_·2H_2_O react under conditions of crystal growth by layering to produce the expected 1D-polymer [Zn_2_(μ-OAc)_4_(**22**)]*_n_*. However, only preliminary crystallographic data could be obtained for this compound, and during attempts to grow better single crystals, a few X-ray quality crystals of the 1D-coordination polymer [2{Zn_7_(μ-OAc)_10_(μ_4_-O)_2_(**22**)}]*_n_*·*n*CH_2_Cl_2_ were obtained. The compound crystallizes in the *P*2_1_2_1_2_1_ space group, and the chiral 1D-chain (Figure 19a) is built up along a 2-fold screw axis. Although crystallization in *P*2_1_2_1_2_1_ space group precludes the presence of crystallographic inversion centers, the {Zn_7_(μ-OAc)_10_(μ_4_-O)_2_} cluster is close to being centrosymmetric. It appears that the packing of chains of the same handedness allows the peripheral naphthyl groups in **22** to be accommodated within a cleft between a pair of 4,2′:6′,4″-tpy units [58]. A layering method was also used to grow single crystals from the reaction between ligand **23** (Scheme 6) and Zn(OAc)_2_·2H_2_O [64]. Both block- and plate-shaped crystals were obtained in the same crystallization tube. Preliminary crystallographic data for the blocks confirmed the assembly of the 1D-polymer [Zn_2_(μ-OAc)_4_(**23**)]*_n_* with a zigzag structure analogous to that shown in Figure 15. However, the plates proved to be an unusual 1D, quadruple-stranded chain containing {Zn_5_(OAc)_10_} units (Figure 19b). The packing of the chains in [Zn_5_(OAc)_10_(**23**)_4_]*_n_*·11*n*H_2_O (Figure 19c) resembles that of the single zigzag chains (Figure 15). PXRD analysis of the bulk material revealed that the dominant product was [Zn_5_(OAc)_10_(**23**)_4_]*_n_*·11*n*H_2_O, rather than the more typical single chain [Zn_2_(μ-OAc)_4_(**23**)]*_n_*. However, the reasons for this remain obscure.

The discussion in this section has revealed that, although {Zn_2_(μ-OAc)_4_} paddle-wheel building blocks are ubiquitous [65] and are ideally suited for connecting divergent 4,2′:6′,4″-tpy metal-binding domains into 1D-coordination polymers, crystallization conditions may lead to competing assemblies. These may involve the incorporation of Zn(OAc)_2_ units with monodentate acetato ligands, or the assembly of Zn*_x_*(OAc)*_y_* clusters. Single products from a reaction cannot be assumed, and we again emphasize the need for routine PXRD to ensure that a single crystal structure is representative of the bulk crystalline material.

### 3.4. Assemblies Containing Zinc(II) and 4,2′:6′,4″-tpy Ligands Functionalized in the 4′-Position with Carboxylates

**Scheme 10:**
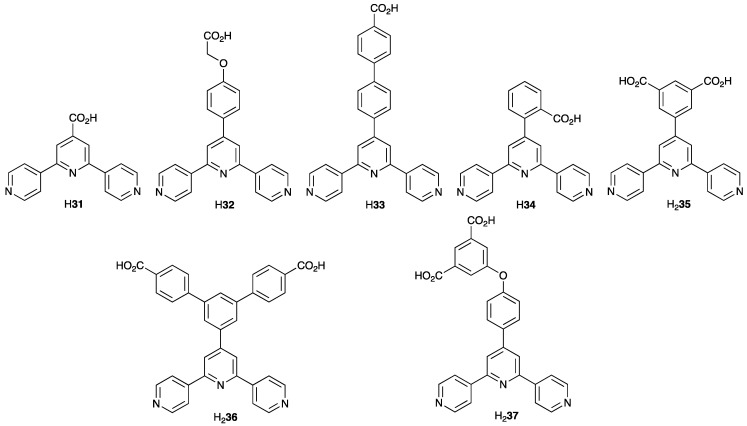
Structures of 4,2′:6′,4″-tpy ligands functionalized in the 4′-position with substituents containing carboxylic acid moeities. See Scheme 6 for the structure of H**17**.

In Section 3.2, we described the structure of the 1D-coordination polymer [Zn_2_(H**17**)_2_Cl_4_]*_n_* [48]. Rather atypically, the carboxylic acid group in H**17** (Scheme 6) remains protonated upon coordination. In other coordination assemblies involving H**17** (see below) and those with ligands H**31**–H**34** and H_2_**35**–H_2_**37** (Scheme 10), deprotonation leads to 4,2′:6′,4″-tpy ligands with carboxylate coordination sites. As we shall see, the presence of an anionic carboxylate donor often leads to preferential coordination of the latter with respect to metal-binding by pyridine donors, with the result that a significant number of assemblies feature pendant, non-coordinated pyridine units.

**Figure 20:**
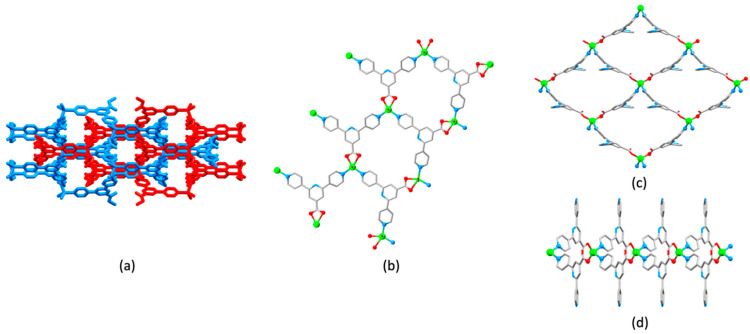
(**a**) Part of the 2-fold interpenetrating 3D-nets in [Zn_2_(**31**)_2_(O_2_CH)_2_]*_n_*·3.5*n*H_2_O (CSD refcode KEPKEY). (**b**) Part of the 2D, 6^3^ net in [{Zn_2_(**31**)_2_(OH_2_)_4_}*_n_*]·[MeCO_2_]_2*n*_·*n*H_2_O (refcode KEPKOY). Parts of the (4,4) net in [Zn(**31**)_2_]*_n_*·*n*H_2_O (refcode KEPLAV) viewed (**c**) down the crystallographic *c*-axis, and (**d**) down the *a*-axis, i.e., into the plane of the 2D-sheet.

**Figure 21:**
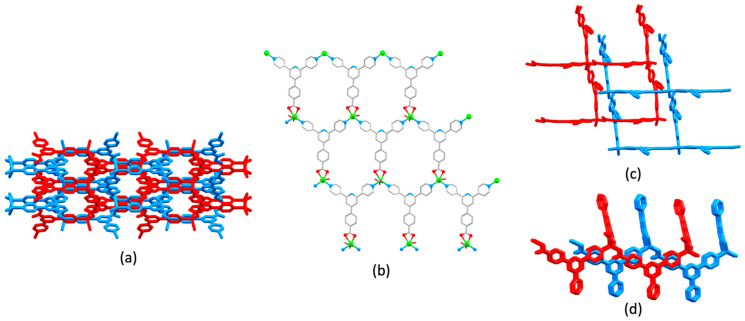
(**a**) Part of the 2-fold interpenetrating 3D-nets in [Zn(**31**)Cl]*_n_*·*n*H_2_O (CSD refcode XEXPUN); note the similarity with Figure 20a. (**b**) Part of the 2D-net found in [{Zn_3_(**31**)_3_(NO_3_)_2_(OH_2_)_4_}*_n_*][NO_3_]*_n_*·5*n*DMF·2*n*H_2_O (refcode DIDNUC). (**c**) Interpenetrated 2D-nets in [Zn_2_(**17**)_4_]*_n_*·0.875*n*H_2_O (CSD refcode GEYTEL); (**d**) the same structure was determined for [Zn_2_(**17**)_4_]*_n_*·*n*H_2_O (CSD recode JEYYUK).

The reaction of zinc(II) acetate and H**31** (Scheme 10) under solvothermal conditions (H_2_O/DMF, pH 4.0, 140 °C) resulted in the formation of [Zn_2_(**31**)_2_(O_2_CH)_2_]*_n_*·3.5*n*H_2_O, with the formato ligand being derived from hydrolysis of DMF. Adjusting the ratio of solvents from 4:1 to 3:2, and raising the pH to 4.5 switches the assembly to [Zn_2_(**31**)_2_(O_2_CH)_2_(OH_2_)_2_]*_n_*·*n*H_2_O. In contrast, keeping the original conditions but replacing DMF by DMA (DMA = dimethylacetamide) led to [{Zn_2_(**31**)_2_(OH_2_)_4_}*_n_*]·[MeCO_2_]_2*n*_·*n*H_2_O. A further change in solvent ratio (H_2_O:DMA = 7:3) and an increase in temperature to 160 °C resulted in crystals of [Zn_2_(**31**)_2_(O_2_CMe)_2_]*_n_*·*n*H_2_O. [Zn_2_(**31**)_2_(O_2_CH)_2_]*_n_*·3.5*n*H_2_O and [Zn_2_(**31**)_2_(O_2_CMe)_2_]*_n_*·*n*H_2_O possess similar 2-fold interpenetrating 3D-nets, and Figure 20a depicts part of the [Zn_2_(**31**)_2_(O_2_CH)_2_]*_n_* network. Each Zn(II) center is tetrahedrally sited, being bound by two *N*-donors from two different **31**^–^ ligands, one carboxylate group from a **31**^–^ ligand, and one monodentate formato ligand. In [Zn_2_(**31**)_2_(O_2_CMe)_2_]*_n_*·*n*H_2_O, the acetato ligand is classed as bidentate (Zn–O = 2.037(5) and 2.482(7) Å). Both [Zn_2_(**31**)_2_(O_2_CH)_2_(OH_2_)_2_]*_n_*·*n*H_2_O and [{Zn_2_(**31**)_2_(OH_2_)_4_}*_n_*]·[MeCO_2_]_2*n*_·*n*H_2_O feature 2D-nets with 6^3^ (*hcb*) topology (Figure 20b). Each net is essentially planar with axial H_2_O, or H_2_O and HCO_2_^–^ ligands decorating the upper and lower surfaces of a sheet [66]. Under hydrothermal conditions (pH 4.0, 160 °C), H**31** reacts with ZnSO_4_·7H_2_O, giving single crystals of [Zn(**31**)_2_]*_n_*·*n*H_2_O. This assembly comprises a 2D (4,4) net (Figure 20c) and a noticeable feature is that the 4,2′:6′,4″-tpy unit binds to zinc(II) through only one *N-*donor. Thus, each **31**^–^ ligand is ditopic. In other (4,4) nets, 4,2′:6′,4″-tpy typically acts as a ditopic *N,N′*-linker (see Section 3.7) and, therefore, the presence of the 4′-CO_2_^–^ is a critical factor is switching the coordination mode. Figure 20d shows that in [Zn(**31**)_2_]*_n_*·*n*H_2_O, the uncoordinated terminal pyridyl groups protrude from above or below the sheet. Zhu and coworkers describe the 2D-sheet as being constructed from left- and right-handed helical chains, the chains being connected through Zn(II) centers, to give 2D-sheets based either on left- or right-handed helices [66]. Under hydrothermal conditions at pH 4.0, H**31** reacts with ZnCl_2_ to give crystals of [Zn(**31**)Cl]*_n_*·*n*H_2_O. Phthalic acid was present in the reaction mixture but was not incorporated as a co-ligand. However, in the absence of phthalic acid, no crystalline material was obtained [67]. This phenomenon is seen repeatedly, and we highlight further examples later in the review. A comparison of Figure 21a with Figure 20a illustrates that the 2-fold interpenetrating assembly in [Zn(**31**)Cl]*_n_*·*n*H_2_O mimics that in [Zn_2_(**31**)_2_(O_2_CH)_2_]*_n_*·3.5*n*H_2_O. Both compounds crystallize in the *Pbcn* space groups and the cell dimensions are similar: for [Zn(**31**)Cl]*_n_*·*n*H_2_O (refcode XEXPUN), *a* = 20.452(3), *b* = 7.2803(10), *c* = 21.203(3) Å, and for [Zn_2_(**31**)_2_(O_2_CH)_2_]*_n_*·3.5*n*H_2_O (refcode KEPKEY), *a* = 20.453(3), *b* = 7.6063(13), *c* = 21.826(3) Å.

Ligands H**17** and H**31** (Scheme 6 and Scheme 10) differ in that the former contains a phenylene spacer between the 4,2′:6′,4″-tpy and carboxylic acid domains. Solvothermal conditions (H_2_O/DMA, HNO_3_, 80 °C) were used for the reaction of Zn(NO_3_)_2_·6H_2_O with H**17**, and the product was shown by X-ray diffraction to be [{Zn_3_(**17**)_3_(NO_3_)_2_(OH_2_)_4_}*_n_*][NO_3_]*_n_*·5*n*DMF·2*n*H_2_O [68]. A 2D-net assembles (Figure 21b) and is closely related to that observed in [{Zn_2_(**31**)_2_(OH_2_)_4_}*_n_*]·[MeCO_2_]_2*n*_·*n*H_2_O (Figure 20b). Under different solvothermal conditions (H_2_O/MeCN, HNO_3_, 160 °C), the reaction between Zn(NO_3_)_2_·6H_2_O and H**17** led to [Zn_2_(**17**)_4_]*_n_*·0.875*n*H_2_O, consisting of 2-fold interpenetrating 2D-nets in which ligand **17**^–^ binds to Zn(II) through only one pyridine ring [30]. The same assembly is produced from Zn(NO_3_)_2_·6H_2_O and H**17** under different conditions (H_2_O/DMA, pH 6.0, 180 °C), although it is formulated as [Zn_2_(**17**)_4_]*_n_*·*n*H_2_O [69]. A comparison of the two structures (both in space group *P*–1) reveals them to be isostructural. Cell dimensions for [Zn_2_(**17**)_4_]*_n_*·0.875*n*H_2_O (CSD refcode GEYTEL) are *a* = 15.120(4), *b* = 15.143(4), *c* = 17.514(5) Å, *α* = 69.388(7), *β* = 86.529(8), *γ* = 79.694(9)° [30], while those reported for [Zn_2_(**17**)_4_]*_n_*·*n*H_2_O (CSD recode JEYYUK) are *a* = 15.102(6), *b* = 15.117(6), *c* = 17.397(7) Å, *α* = 86.631(5) *β* = 69.642(4), *γ* = 79.346(5)° [69]. The 2D→2D parallel interpenetration is shown in Figure 21c. Figure 21d illustrates the projection of arene substituents above and below the 2D-sheets, and face-to-face π-stacking between arene rings in adjacent layers locks the assembly together. In [Zn_2_(**17**)_2_Cl_2_]*_n_*·0.5*n*H_2_O (refcode JUHYOC), **17**^–^ behaves in a tritopic manner, and a 6^3^ net assembles, and undergoes 2-fold interpenetration [70].

**Figure 22:**
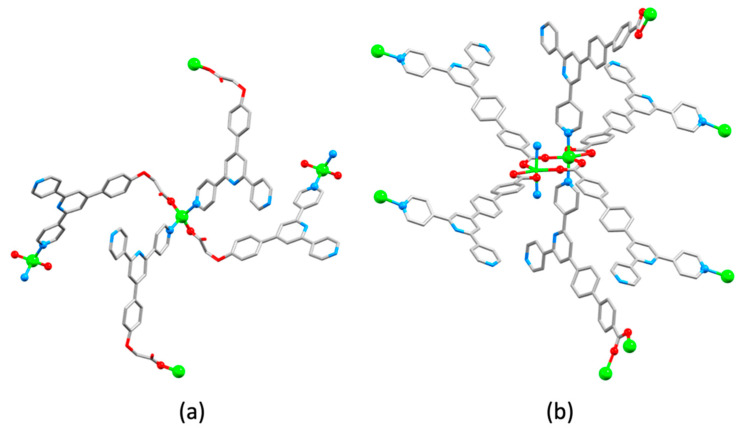
(**a**) Coordination environment of Zn(II) in [Zn(**32**)_2_]*_n_* which leads to the metal center being a 4-connecting node in a *dia* net (CSD refcode ZIDREL). (**b**) Coordination environment around the 8-connecting node in [Zn(**33**)_2_]*_n_* (refcode TEZJOA); for clarity, two of the eight ligand-linkers are represented only with the coordinated nitrogen atom.

In [Zn_2_(**17**)_4_]*_n_*·*n*H_2_O and [Zn_2_(**17**)_4_]*_n_*·0.875*n*H_2_O (Figure 21c,d), the 4,2′:6′,3″-tpy unit was monodentate, and this is also observed in [Zn(**32**)_2_]*_n_* (see Scheme 10 for H**32**). Note that ligands H**17** and H**32** differ only by the introduction of an OCH_2_ unit, and this has a significant effect on the assembly. [Zn(**32**)_2_]*_n_* was prepared under hydrothermal conditions (pH 4.0, 160 °C) and adopts an interpenetrated 4-connected 3D network. Each Zn(II) center is bound by two pyridine rings (from different ligands) and two monodentate carboxylates (from different ligands) as shown in Figure 22a, and the structure propagates into a *dia* network which undergoes 3-fold interpenetration [71]. Upon moving from H**17** and H**32** to H**33** (Scheme 10), the distance between the 4,2′:6′,4″-tpy and carboxylic acid domains increases further; note that H**33** possesses a relatively rigid backbone. The reaction of H**33** with Zn(NO_3_)_2_·6H_2_O under solvothermal conditions (H_2_O/DMF, 120 °C) produced [Zn(**33**)_2_]*_n_*·*xn*H_2_O (*x* was not determined). As in the examples above, the 4,2′:6′,4″-tpy domain in **33**^–^ coordinates only through one pyridine ring, but in contrast to the coordination environment in [Zn(**32**)_2_]*_n_* (Figure 22a), the Zn(II) centers in [Zn(**33**)_2_]*_n_*are dinuclear (Figure 22b). Each unit acts as an 8-connected node (only six ligand linkers are shown in Figure 22b for clarity). The 3D-assembly is described by the authors as a (2,8)-connected network with a topological point symbol of {4^24^·6^4^}; see Section 3.8 for a comparison with the cadmium analog. The MOF was used as a multi-responsive fluorescent sensor for simultaneously detecting Hg^2+^, [CrO_4_]^2−^ and [Cr_2_O_7_]^2−^ ions in aqueous solution with low detection limits [72].

**Figure 23:**
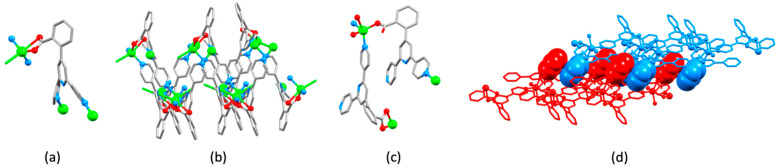
(**a**) The tritopic binding-mode of **34**^–^ in [Zn(**34**)Cl]*_n_* (CSD refcode FIZVUH) and (**b**) part of a 2D-sheet in [Zn(**34**)Cl]*_n_*. (**c**) The two crystallographically independent **34**^–^ linkers in [Zn_2_(**34**)_4_]*_n_*·2*n*H_2_O (refcode FIZWAO), and (**d**) π-stacking between phenyl rings in adjacent 2D sheets in [Zn_2_(**34**)_4_]*_n_*·2*n*H_2_O.

Hu and coworkers have reported a series of six zinc(II)-containing assemblies with the conjugate base of H**34** (Scheme 10) [73]. Crystals were all grown under hydrothermal conditions (180 °C), and we include here only those structures in which there is no co-ligand. The reaction of ZnCl_2_ and H**34** gave [Zn(**34**)Cl]*_n_*, while with Zn(OAc)_2_·2H_2_O, crystals of [Zn_2_(**34**)_4_]*_n_*·2*n*H_2_O were obtained. It is relevant to compare the structures of these assemblies with those containing **17**^–^, because linkers **17**^–^ and **34**^–^ are isomers. We described earlier that in [Zn_2_(**17**)_2_Cl_2_]*_n_*·0.5*n*H_2_O [70], the ligand bound three Zn centers and assembled a 6^3^ net. In [Zn(**34**)Cl]*_n_*, the ligand is again tritopic (Figure 23a), but the differing vectorial properties of the donor atoms compared to those in **17**^–^ lead to a 2D-sheet architecture which the authors describe in terms of 1D-loop chains, which are further linked by a terminal nitrogen atom and carboxylate group. As Figure 23b illustrates, the structure is not easy to envisage. [Zn_2_(**34**)_4_]*_n_*·2*n*H_2_O is also a 2D-network, but in this assembly, **34**^–^ is ditopic and coordinates through one pyridine ring and the carboxylate group. Figure 23c shows the coordination modes of the two independent ligands. The structure propagates into a 2D sheet, and the phenyl rings are involved in face-to-face contacts between the layers (Figure 23d). Figure 23 shows that a feature common to both [Zn(**34**)Cl]*_n_* and [Zn_2_(**34**)_4_]*_n_*·2*n*H_2_O is that the arene rings of the 2-carboxyphenyl groups are located on the upper and lower surfaces of the 2D-nets.

**Figure 24:**
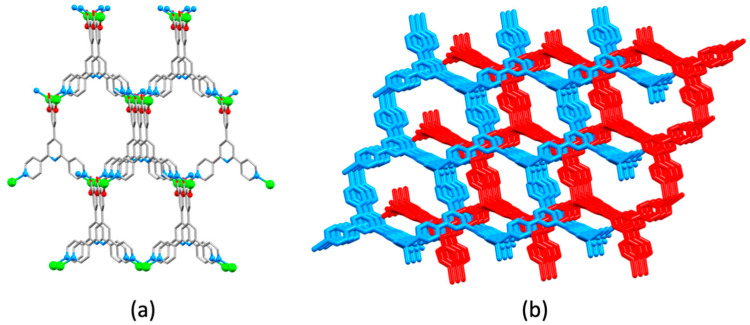
(**a**) Part of one 3D-network in [Zn(**35**)]*_n_* with both Zn(II) and the ligand acting at 4-connecting nodes (CSD refcode HOSMEK). The nets in QIDQUS and WUWBUO are identical. (**b**) The 2-fold interpenetration of 3D-nets in [Zn(**35**)]*_n_*.

**Figure 25:**
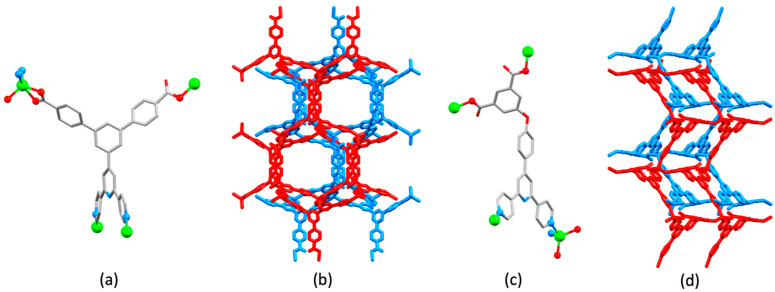
(**a**) The tetratopic binding-mode of **36**^2–^ in [Zn(**36**)]*_n_*·4*n*DMF (CSD refcode QOZSIJ) and (**b**) the 2-fold interpenetration of *lon*-nets in [Zn(**36**)]*_n_*·4*n*DMF. (**c**) The **37**^2–^ ligand is a 4-connecting node in [Zn(**37**)]*_n_* (refcode SAHWEG). (**d**) Part of the 2-fold interpenetrating 3D-nets in [Zn(**37**)]*_n_* viewed down the crystallographic *c*-axis.

Dicarboxylic acids H_2_**35**, H_2_**36** and H_2_**37** (Scheme 10) all act as tetratopic ligands with zinc(II). Feng and coworkers have described the solvothermal reaction of H_2_**35** with Zn(NO_3_)_2_·6H_2_O (DMA, 120 °C) leading to crystals of [Zn(**35**)]*_n_*·*n*DMA·2*n*H_2_O [74]. The compound (refcode WUWBUO) crystallizes in the triclinic *P*–1 space group and cell dimensions are identical to those of [Zn(**35**)]*_n_*·1.25*n*H_2_O and [Zn(**35**)]*_n_*·4.17*n*H_2_O, the structures of which have been deposited to the CSD as communications [75,76]. Both the Zn(II) center and **35**^2–^ ligand in [Zn(**35**)]*_n_* are 4-connecting nodes, and both are distorted tetrahedral. Figure 24a illustrates part of the 3D-network (with *dia* topology) that is assembled; the view highlights the hexagonal channels running parallel to the *a*-axis. Two-fold interpenetration occurs as shown in Figure 24b. On going from H_2_**35** to H_2_**36**, additional phenylene spacers are introduced (Scheme 10). The solvothermal reaction of H_2_**36** with Zn(NO_3_)_2_·6H_2_O (DMF, 100 °C) yielded crystals of [Zn(**36**)]*_n_*·4*n*DMF [77]. As in [Zn(**35**)]*_n_*, both Zn(II) and **36**^2–^ in [Zn(**36**)]*_n_*·4*n*DMF are 4-connecting, approximately tetrahedral nodes (Figure 25a). However, in this case a *lon* net assembles, with 2-fold interpenetration (Figure 25b). The *lon* net includes boat-form six-membered rings which are not present in a *dia* net [78]. The activated [Zn(**36**)]*_n_* MOF has high porosity with a micropore volume of 0.89 m^3^ g^–1^. In addition, [Zn(**36**)]*_n_*·4*n*DMF exhibits efficient luminescence sensing for Cu^2+^ ions [77]. The conformational flexibility of H_2_**37** (Scheme 10) contrasts with that of H_2_**35** and H_2_**36**. The reaction of H_2_**37** with Zn(NO_3_)_2_·6H_2_O under solvothermal conditions (MeCN/H_2_O/NaOH, 140 °C) produced crystals of solvated [Zn(**37**)]*_n_*. Both the ligand (Figure 25c) and Zn(II) center are 4-connecting nodes. A complex 3D-net assembles and, as in the previous examples, the large voids present in the net, lead to 2-fold interpenetration. In common with related MOFs, the presence of solvent-accessible voids coupled with the non-coordinated pyridine ring of the 4,2′:6′,4″-tpy domain make [Zn(**37**)]*_n_* a candidate for fluorescence sensing for both metal cations and small molecules [79].

### 3.5. Assemblies Containing Zinc(II) and 4,2′:6′,4″-tpy Ligands Functionalized in the 4′-Position with a Pyridinyl Group or Other Nitrogen Heterocycles

**Scheme 11:**
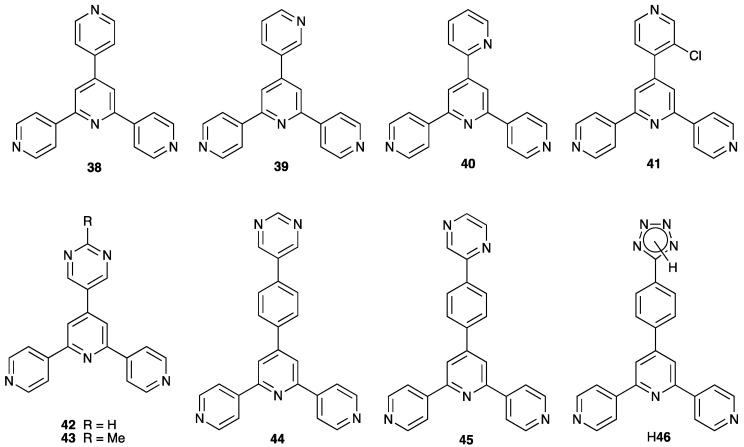
Structures of 4,2′:6′,4″-tpy ligands functionalized in the 4′-position with a pyridinyl group or other nitrogen heterocycles.

The introduction into a 4,2′:6′,4″-tpy unit of an additional nitrogen-containing heterocyclic substituent is a popular strategy for increasing the dimensionality of a coordination assembly. However, just as we have noted that the 4,2′:6′,4″-tpy may bind in either a mono- or ditopic manner, it is difficult to predict whether the added heterocyclic substituent will be coordinatively active or innocent. Scheme 11 summarizes the ligands discussed in this section; note that ligands **38**, **39** and **40** are isomers of each other.

**Figure 26:**
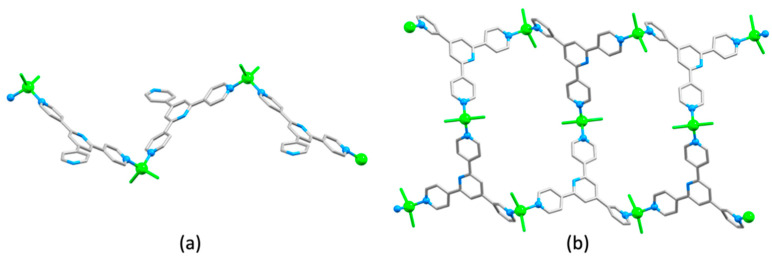
(**a**) Part of the 1D-polymer in [Zn(**38**)Cl_2_]*_n_* (CSD refcode AGUPEY), showing non-coordinated 4′-(pyridin-4yl) substituents. (**b**) Part of 1D-ladder in [Zn_3_(**39**)_2_Cl_6_]*_n_*·*n*H_2_O (refcode ZOKFAI).

Ligand **38** appears both as a ditopic and tritopic building block in coordination assemblies with zinc(II). Reactions with zinc(II) halides yield varying products, and whether this is a consequence of reactions conditions or serendipity is difficult to assess in the absence of PXRD data for bulk materials. Hu and coworkers reported that, under hydrothermal conditions (160 °C), ZnCl_2_·2H_2_O and **38** react to give single crystals of [Zn(**38**)Cl_2_]*_n_*. The structure is a helical 1D-coordination polymer with pendant, non-coordinated pyridin-4-yl substituents (Figure 26a) [80]. In contrast, Dehnen and coworkers observed the assembly of interlocked molecular cages in [Zn_12_(**38**)_8_Cl_24_]*_n_*·*n*CHCl_3_ when crystals were grown by layering a chloroform solution of **38** over a methanol solution of ZnCl_2_ [81]. Dehnen reported that a change to ZnI_2_ resulted in the assembly of a 1D-chain [81], but in contrast, we have observed that reacting ZnI_2_ with **38** under conditions of crystal growth by layering resulted in the assembly of polycatenated icosahedral [Zn_12_(**38**)_8_I_24_] cages [59], analogous to those in [Zn_12_(**38**)_8_Cl_24_]*_n_*·*n*CHCl_3_ [81]. Retaining a layering technique, we also demonstrated that the introduction of a chloro-substituent on going from ligand **38** to **41** (Scheme 11) affects the outcome of the reaction with ZnI_2_. Instead of the assembly of an [Zn_12_(**38**)_8_I_24_] cage, crystals of the 1D-polymer [Zn(**41**)I_2_]*_n_*·0.25*n*H_2_O were isolated. A combination of **41** and zinc(II) chloride also gave a 1D-coordination polymer, [Zn(**41**)Cl_2_]*_n_* [59].

We saw earlier that, under hydrothermal conditions, Hu and coworkers assembled [Zn(**38**)Cl_2_]*_n_* with a simple 1D-polymer assembly (Figure 26a) [80]. An interesting change occurs when moving from **38** to its isomer **39**. Again, under solvothermal conditions, ZnCl_2_ reacts with **39** to produce [Zn_3_(**39**)_2_Cl_6_]*_n_*·*n*H_2_O. This consists of a 1D-coordination polymer in which **39** acts as a tritopic ligand, leading to a ladder-type of assembly (Figure 26b) [82]. Unfortunately, there is no example of a coordination assembly featuring the third ligand isomer **40** with a zinc(II) halide, so no direct comparisons are possible. However, a comparison can be made between the structures of the coordination polymers formed in the reactions of Zn(OAc)_2_·2H_2_O and ligands **38** or **40**. Zhang et al. reported that crystal growth by layering at room temperature led to the assembly of [Zn_2_(μ-OAc)_4_(**38**)]*_n_*·1.5*n*MeOH and [Zn(OAc)_2_(**40**)]*_n_* [61]. Both contain 1D-chains, and the 4′-pyridinyl substituent is non-coordinated. The assembly in [Zn_2_(μ-OAc)_4_(**38**)]*_n_*·1.5*n*MeOH falls into the category of the structures described in Section 3.3, while [Zn(OAc)_2_(**40**)]*_n_* possesses a helical 1D-polymer. In both crystal growth experiments, a ratio of Zn:ligand of 2:1 was used, and the difference in the assemblies is not unique (see Table 2 and discussion) but is not easily explained.

**Figure 27:**
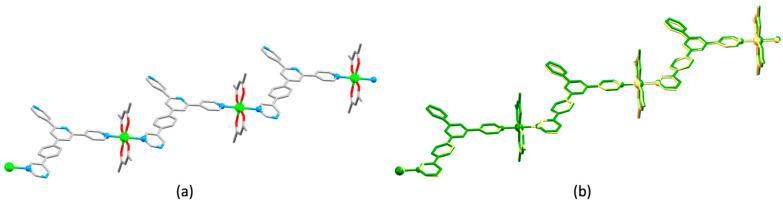
(**a**) Part of the 1D-chain in [Zn(**44**)(acac)_2_]*_n_* (CSD refcode QIXYON) showoing coordination to Zn(II) through pyrimidine and pyridine donors. (**b**) Overlay of parts of the polymers in [Zn(**44**)(acac)_2_]*_n_* (shown in cream) and [Zn(**45**)(acac)_2_]*_n_*(in green, refcode QIXYON01).

The pyrimidin-5-yl functionalities in ligands **42** and **43** remain non-coordinated in the 1D-coordination polymers [Zn(**42**)Cl_2_]*_n_*, [Zn(**42**)I_2_]*_n_* and [Zn(**43**)I_2_]*_n_*·*n*MeOH [83]. In contrast, Granifo et al. reported that **44** combined with Zn(acac)_2_ (Hacac = pentane-2,4-dione) in a preparative scale reaction to give a mixture of needle-like and block-like crystals. These were separated by hand, and structural analysis confirmed the assembly of the mononuclear complex [Zn(**44**)_2_(acac)_2_] (in which **44** is monodentate, coordinating through one pyridine ring) and a coordination polymer [Zn(**44**)(acac)_2_]*_n_* (Figure 27b) [84]. The coordination mode of **44** is atypical since, although the ligand is ditopic, the preference is for coordination through one pyridine and one pyrimidine ring rather than through both the outer pyridine rings of the 4,2′:6′,4″-tpy unit. The formation of [Zn(**44**)_2_(acac)_2_] and [Zn(**44**)(acac)_2_]*_n_* is yet another example of competition between assemblies formed under the same synthetic conditions, and illustrates once again, the difficulties in predictive crystal engineering with these families of ligands. Granifo and coworkers have also prepared **45** in which a pyrazin-2-yl group replaces the pyrimidin-5-yl functionality in **44**. The coordination behavior of **45** in [Zn(**45**)(acac)_2_]*_n_* [85] mimics that of **44** in [Zn(**45**)(acac)_2_]*_n_* as can be seen from the overlay of the two polymer chains in Figure 27b.

**Figure 28:**
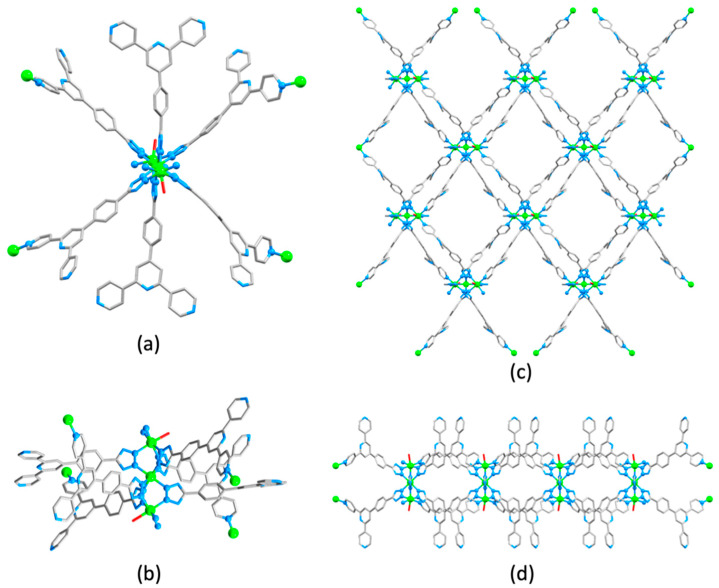
The structure of [Zn_3_(**46**)_6_(OH_2_)_2_]*_n_*·2H_2_O (CSD refcode NUBZUI). Two views of the {Zn_3_(**46**)_6_(OH_2_)_2_} building block showing (**a**) four coordinating and two non-coordinating 4,2′:6′,4″-tpy units, and (**b**) the {Zn_3_(μ-triazolato)_6_} node. Part of one 2D-network, viewed (**c**) down the crystallographic *c*-axis, and (**d**) down the *a*-axis; the non-coordinated 4,2′:6′,4″-tpy units with their phenylene spacers are omitted for clarity.

The tetrazole-functionalized compound H**46** (Scheme 11) binds zinc(II) to form [Zn_3_(**46**)_6_(OH_2_)_2_]*_n_*·2H_2_O No synthetic details are available for this CSD communication [86]. [Zn_3_(**46**)_6_(OH_2_)_2_]*_n_*·2H_2_O crystallizes in the monoclinic space group *C*2/*c*, and Figure 28a,b displays two views of the basic building block. Central to this is a linear Zn_3_ core, with each Zn…Zn vector bridged by three triazolato-units. Of the six 4,2′:6′,4″-tpy domains associated with this trinuclear core, four bind to Zn(II) centers in neighboring Zn_3_ units, and two are non-coordinating (Figure 28a). The assembly propagates into a 2D-network, as illustrated in Figure 28c,d. Although the architecture is unusual in terms of the coordination mode of the 4,2′:6′,4″-tpy domains, it is pertinent to note that a search of the CSD [16] revealed that the {Zn_3_(μ-triazolato)_6_} unit is well represented, and it is therefore likely that the assembly of this motif directs the assembly in [Zn_3_(**46**)_6_(OH_2_)_2_]*_n_*·2H_2_O.

### 3.6. An Assembly Containing Zinc(II) and a 4,2′:6′,4″-tpy Ligand Functionalized in the 4′-Position with an Oxygen Heterocycle

**Figure 29:**
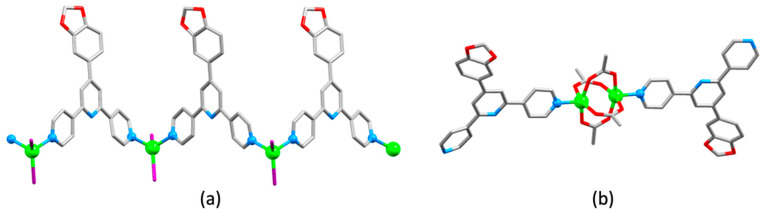
(**a**) Part of the 1D-polymer in [Zn(**47**)I_2_]*_n_* (CSD refcode QOTSAV), and (**b**) the structure of the discrete complex [Zn_2_(μ-OAc)_4_(**47**)_2_] (refcode QOTRUO).

4′-(1,3-Benzodioxol-5-yl)-4,2′:6′,4″-terpyridine (**47**) appears to be the sole example of a 4,2′:6′,4′′-tpy bearing a 4′-substituent with an *O*-heterocyclic unit. Its reactions with ZnI_2_ and Zn(OAc)_2_·2H_2_O under ambient conditions of crystal growth by layering, led to crystals of [Zn(**47**)I_2_]*_n_* and [Zn_2_(μ-OAc)_4_(**47**)_2_], respectively. The iodido complex is a 1D-coordination polymer with the chain (Figure 29a) built up by a glide plane. In contrast, [Zn_2_(μ-OAc)_4_(**47**)_2_] is a discrete molecular complex (Figure 29b), again illustrating a 4,2′:6′,4′′-tpy acting as a monodentate ligand. Little can be concluded about the differences in the assemblies, since the PXRD for the bulk material for [Zn(**47**)I_2_]*_n_* indicates the presence of another phase, and PXRD data could not be obtained for the bulk sample of [Zn_2_(μ-OAc)_4_(**47**)_2_] because of decomposition in air [61].

### 3.7. Assemblies Containing Cadmium(II) and 4′-Functionalized 4,2′:6′,4″-tpy Ligands with Coordinatively Innocent Substituents

**Scheme 12:**
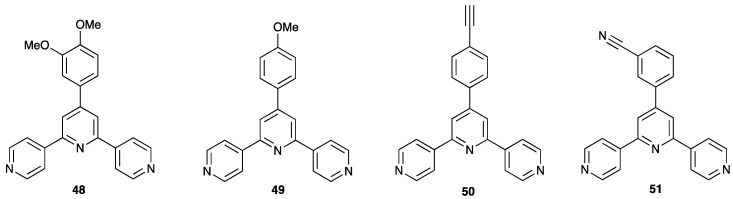
Structures of some ligands with non-coordinating 4′-functionalities that form coordination assemblies with cadmium(II). Other relevant ligands are **14** (Scheme 6), **28**, **29** and **30** (Scheme 9).

We now move to cadmium(II) coordination polymers containing 4,2′:6′,4″-tpy linkers. No structurally characterized example with a simple 4,2′:6′,4″-tpy has been reported, although assemblies with coordinatively innocent substituents in the 4′-position of 4,2′:6′,4″-tpy are well represented. Scheme 12 summarizes some of the ligands referred to in this section.

**Figure 30:**
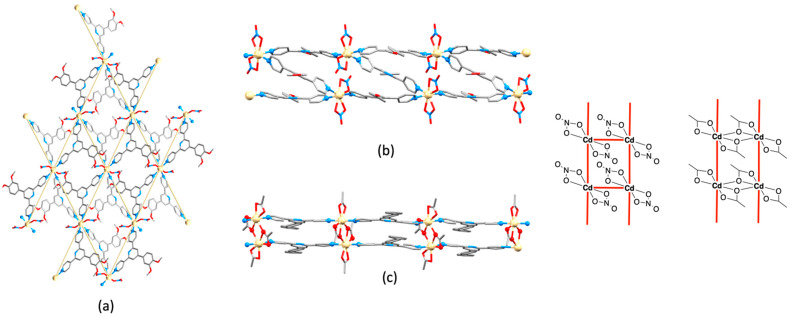
(**a**) Part of one (4,4) net in [Cd(**48**)_2_(NO_3_)_2_]*_n_* (CSD refcode COGWEC); each Cd atom is a 4-connecting node and the rhombuses in the net are highlighted. Part of the 1D-ladder (**b**) in [Cd_2_(**49**)_3_(NO_3_)_4_]*_n_·n*MeOH***^.^****n*CHCl_3_ (CSD refcode BUDZIL), and (**c**) [Cd_2_(OAc)_4_(**30**)_2_]*_n_·*1.75*n*MeOH·0.5*n*H_2_O (CSD refcode RIGJEY). Right: a schematic representation of the different ladder assemblies.

**Figure 31:**
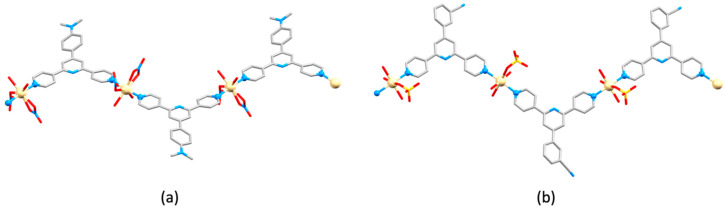
Part of the 1D-ladder (**a**) in [Cd(**29**)(OH_2_)_2_(NO_3_)_2_]*_n_*·*n*H_2_O (CSD refcode VAKWOV) and in (**b**) [Cd_2_(**51**)_2_(SO_4_)_2_(H_2_O)_6_]*_n_*·*n*H_2_O (refcode NUNZON).

Crystal growth by layering has been used for the assembly of [Cd(**14**)_2_(NO_3_)_2_]*_n_·n*MeOH***^.^****n*CHCl_3_ [87], which consists of a (4,4) net with Cd(II) acting as a 4-connecting node. This contrasts with the numerous 1D-polymers that are observed for combinations of zinc(II) salts with 4,2′:6′,4″-tpy ligands with non-coordinating substituents, and highlights the fact that the higher coordination numbers favored by Cd(II) can lead to higher dimensionality assemblies. Similar (4,4)-nets are formed when Cd(NO_3_)_2_·4H_2_O, CdBr_2_ or CdI_2_ reacts with **48** (Figure 30a) [88], or when Cd(NO_3_)_2_·4H_2_O combines with 4′-(4-ROC_6_H_4_)-4,2′:6′,4″-tpy in which R = *^n^*pentyl, *^n^*hexyl, or *^n^*heptyl [89]. However, for smaller R groups, the assembly switches to either a ladder (for R = Me, ligand **49**) [90] or a (6,3) net (for R = *^n^*propyl) [89]. The ladder in [Cd_2_(**49**)_3_(NO_3_)_4_]*_n_·n*MeOH***^.^****n*CHCl_3_ is of interest because ditopic 4,2′:6′,4″-tpy units comprise both the rungs and the rails of the ladder (Figure 30b). This is also observed in [Cd_2_(**28**)_3_(NO_3_)_4_]*_n_·*4*n*MeOH·2*n*CHCl_3_ [91], and [Cd_2_(**50**)_3_(NO_3_)_4_]*_n_·*2*n*MeOH·3*n*CHCl_3_·*n*H_2_O [91]. In contrast, when Cd(OAc)_2_·H_2_O is used in place of the nitrate salt, the tendency for dinuclear {Cd_2_(OAc)_4_} units to form directs the assembly to a ladder in which each rung is built from a {Cd_2_(OAc)_4_} group and the 4,2′:6′,4″-tpy linkers form the rails (Figure 30c) [62]. This latter polymer, [Cd_2_(OAc)_4_(**30**)_2_]*_n_·*1.75*n*MeOH·0.5*n*H_2_O, also incorporates 4′-biphenyl substituents, and the structure shown in Figure 30c illustrates that face-to-face π-stacking of biphenyl groups within a chain occurs. To what extent this also helps to direct the assembly is difficult to assess. We noted above that, when compared to Zn(II), the higher coordination numbers typically found for Cd(II) may be a means of accessing higher dimensionality structures. However, there are always examples to underline the dangers of ‘rule making′. Under ambient conditions, ligand **29** (Scheme 6) reacted with Cd(NO_3_)_2_·4H_2_O to give crystals of the 1D-coordination polymer [Cd(**29**)(OH_2_)_2_(NO_3_)_2_]*_n_*·*n*H_2_O (Figure 31a) [91]. The coordinated aqua ligands block sites for extension of the assembly into a 2D-sheet (e.g., as in [Cd_2_(**28**)_3_(NO_3_)_4_]*_n_*, see above). The zig-zag chains in [Cd(**29**)(OH_2_)_2_(NO_3_)_2_]*_n_*·*n*H_2_O nest into one another to form 2D-sheets, and the latter pack in a similar manner to the [Zn_2_(OAc)_4_(4′-X-4,2′:6′,4″-tpy)]*_n_* chains described in Figure 15 and Figure 16. The 1D-polymer [Cd_2_(**51**)_2_(SO_4_)_2_(H_2_O)_6_]*_n_*·*n*H_2_O assembled in the reaction of CdSO_4_ with ligand **51** (Scheme 12) under solvothermal conditions (EtOH/H_2_O, 120 °C). As in [Cd(**29**)(OH_2_)_2_(NO_3_)_2_]*_n_*, the *trans*-arrangement of coordinated 4,2′:6′,4″-tpy units leads to a zig-zag chain in [Cd_2_(**51**)_2_(SO_4_)_2_(H_2_O)_6_]*_n_* (Figure 31b).

### 3.8. Assemblies Containing Cadmium(II) and 4,2′:6′,4″-tpy Ligands Functionalized in the 4′-Position with Carboxylate or Sulfonate Donors

In Section 3.4, we reviewed coordination polymers and networks derived from reactions of zinc(II) salts and 4,2′:6′,4″-tpy ligands functionalized with carboxylate donors. We now turn to assemblies combining some of the same ligands (H**17**, H**31**, H**33**, H**34** and H_2_**37**) with cadmium(II). The structures of these ligands are given in Scheme 6 and Scheme 10.

**Figure 32:**
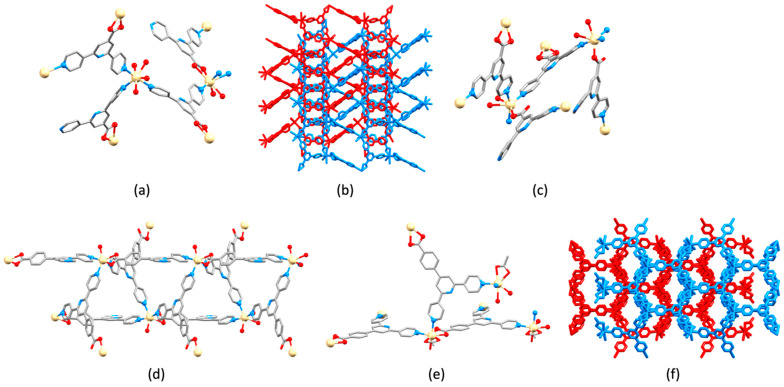
(**a**) The Cd(II) coordination environments and ligand coordination modes in [Cd_2_(**31**)_4_]*_n_* (CSD refcode KEPLID), and (**b**) the 2-fold interpenetrated 3D-network in [Cd_2_(**31**)_4_]*_n_*. (**a**,**c**) The Cd(II) coordination environments and ligand coordination modes in [Cd_2_(**31**)_4_]*_n_*·2*n*H_2_O (CSD refcode XIDCEU). (**d**) Part of the 2D-net in [Cd(**17**)_2_]*_n_*·2*n*DMF (refcode HEKTEY). (**e**) In [Cd(**17**)(OAc)(OH_2_)]*_n_*·*n*DMA·*n*H_2_O (CSD refcode HEKTAU), both the Cd(II) center and **17**^–^ ligand are 3-connecting nodes. (**f**) The 2-fold interpenetrating *ths* nets in [Cd(**17**)(OAc)(OH_2_)]*_n_*·*n*DMA·*n*H_2_O.

Carboxylic acids H**31**, H**17**, and H**33** differ in having no, one and two phenylene spacers, respectively, between the 4,2′:6′,4″-tpy and carboxylic acid domains. The solvothermal reaction (DMF/H_2_O, pH 4.0, 160 °C) of CdBr_2_ and H**31** produced crystals of [Cd_2_(**31**)_4_]*_n_* [66]. The compound crystallizes in the monoclinic space group *P*2_1_/*n* and the asymmetric unit contains two crystallographically independent ligands and two independent Cd(II) centers. Both metal centers are bound by three *N*-donors of three different **31**^–^ ligands and by two bidentate carboxylates from two different ligands, making each Cd(II) center a 5-connecting node (Figure 32a). The coordination behaviors of the 4,2′:6′,4″-tpy units in the two independent **31**^–^ ligands differ in that one coordinates through both outer pyridine rings, and the second through only one (Figure 32a). Thus, every other **31**^–^ ligand is a 3-connecting node, and the remaining ligands are purely linkers. The structure extends into a (3,5)-connected 3D-network, and is 2-fold interpenetrating (Figure 32b). A comparison should be made between this structure and that of [Zn(**31**)_2_]*_n_*·*n*H_2_O (Figure 20d), and the effects of the higher coordination number of Cd(II) versus Zn(II) are clear. In addition to [Zn(**31**)_2_]*_n_*·*n*H_2_O and [Cd_2_(**31**)_4_]*_n_*, Zhou and coworkers have also investigated the consequences of introducing a series of co-ligands [66]. Hu and coworkers have reported [Cd_2_(**31**)_4_]*_n_*·2*n*H_2_O, which was formed under hydrothermal conditions (pH 7.5, 160 °C) in the presence of oxalic acid. Although oxalate is not incorporated into the assembly as a coligand, the presence of oxalic acid in the reaction mixture was essential for the growth of single crystals of [Cd_2_(**31**)_4_]*_n_*·2*n*H_2_O [92]. This is reminiscent of separate observations from the same group that, under hydrothermal conditions, single crystals of [Zn(**31**)Cl]*_n_*·*n*H_2_O could only be obtained if phthalic acid were present [67]. [Cd_2_(**31**)_4_]*_n_*·2*n*H_2_O crystallizes in the orthorhombic space group *Pbcn*, making it structurally distinct from [Cd_2_(**31**)_4_]*_n_* described above. However, a comparison of Figure 32a and c reveals that in both [Cd_2_(**31**)_4_]*_n_* and the dihydrate, one ligand **31**^–^ acts as a 3-connecting node, and one ligand bridges two Cd(II) centers. The assembly in [Cd_2_(**31**)_4_]*_n_*·2*n*H_2_O is, like, [Cd_2_(**31**)_4_]*_n_*, a 2-fold interpenetrating, (3,5)-connected 3D-network [92].

**Figure 33:**
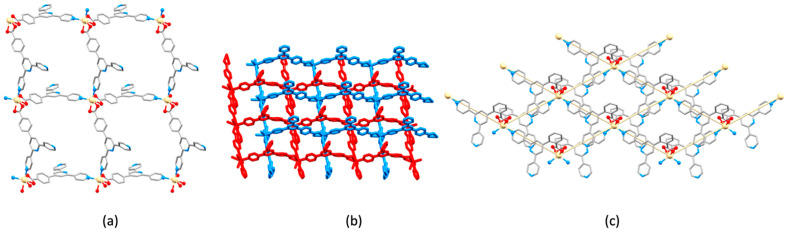
(**a**) Part of one 2D-sheet in [Cd_2_(**17**)_4_]*_n_*·3.5*n*H_2_O (CSD refcode JUHYUI), and (**b**) 2D→2D parallel interpenetration of the nets in this assembly. (**c**) Part of the 2D-net in [Cd_2_(**34**)_4_]*_n_*·*n*H_2_O with rhombuses highlighted (refcode FIZWES).

Ligand H**17** and H**34** are isomers (Scheme 6 and Scheme 10). [Cd(**17**)_2_]*_n_*·2*n*DMF and [Cd(**17**)(OAc)(OH_2_)]*_n_*·*n*DMA·*n*H_2_O were formed under solvothermal conditions using Cd(OAc)_2_·2H_2_O and H**17** in DMA/H_2_O, or Cd(NO_3_)_2_·4H_2_O and H**17** in DMF/H_2_O with, in the latter case, 4,4′-oxybis(benzoic acid) added, presumably, as a potential coligand. Consistent with observations described above, crystal growth was dependent upon the presence of this additional component, suggesting a possible templating role. The structure of [Cd(**17**)_2_]*_n_*·2*n*DMF (which crystallizes in the monoclinic *C*2/*c* space group) is a (3,5)-connected 2D network, part of which is displayed in Figure 32d. This contrasts with the interpenetrated (3,5)-connected 3D nets in [Cd_2_(**31**)_4_]*_n_*·2*n*H_2_O and [Cd_2_(**31**)_4_]*_n_*, and the change in assembly appears to be associated with the introduction of the phenylene spacer in **17**^–^. As in [Cd_2_(**31**)_4_]*_n_*·2*n*H_2_O and [Cd_2_(**31**)_4_]*_n_*, there are two crystallographically independent ligands in [Cd(**17**)_2_]*_n_*·2*n*DMF. One acts as a 3-connecting node, and one coordinates only through one pyridine ring and the CO_2_^–^ unit [93]. In [Cd(**17**)(OAc)(OH_2_)]*_n_*·*n*DMA·*n*H_2_O, every **17**^–^ ligand is crystallographically equivalent, as is every Cd(II) center. Each act as a 3-connecting node (Figure 32e), generating a (10–3)-*b* (or *ths*) 3D-net. The large voids in the net lead to 2-fold interpenetration (Figure 32f). The activated forms of [Cd(**17**)(OAc)(OH_2_)]*_n_* and [Cd(**17**)_2_]*_n_* show selective adsorption of CO_2_ over N_2_, and of H_2_O over MeOH [93]. Hu and coworkers reacted H**17** with CdCl_2_ under hydrothermal conditions and obtained single crystals of [Cd_2_(**17**)_4_]*_n_*·3.5*n*H_2_O. Another variation of the coordination mode of **17**^–^ is exemplified in this compound; every ligand is a linker, coordinating through one pyridine *N*-donor and a bidentate carboxylate group. Each Cd(II) center is a 4-connecting node and a (4,4) (or *sql*) net assembles (Figure 33a). As Figure 33b illustrates, 2D→2D parallel interpenetration of the nets occurs to reduce the void space [70]. In Figure 23, we illustrated coordination assemblies formed between Zn(II) and the conjugate base of H**34** (Scheme 10). These networks from the group of Hu [73] are complemented by that of [Cd_2_(**34**)_4_]*_n_*·*n*H_2_O, single crystals of which were grown under hydrothermal conditions (pH 5, 180 °C) from the reaction between CdCl_2_·2.5H_2_O and H**34**. Whereas the analogous reaction between ZnCl_2_ and H**34** gave [Zn(**34**)Cl]*_n_*, the cadmium-containing assembly contains no chlorido ligands. Each **34**^–^ ligand coordinates to one Cd(II) through one pyridine ring and to a second Cd(II) through a bidentate carboxylate group. Each Cd(II) center is a 4-connecting node, leading to the (4,4) net depicted in Figure 33c.

**Figure 34:**
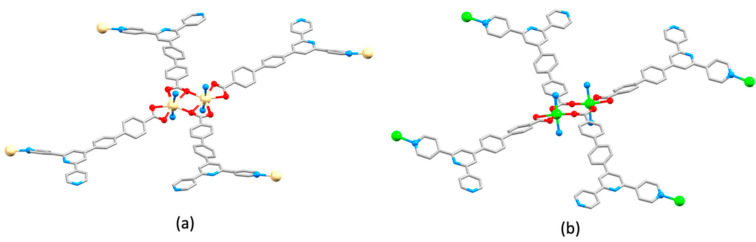
(**a**) The {Cd_2_(O_2_C)_4_} node showing four of the eight coordinated ligands in [Cd(**33**)_2_]*_n_*·*n*DMF·2*n*H_2_O (CSD refcode BOBRIW), compared with (**b**) the corresponding unit in [Zn(**33**)_2_]*_n_* (refcode TEZJOA).

The introduction of an additional phenylene spacer on going from H**17** to H**33** (Scheme 6 and Scheme 10) had a significant influence on the coordination assembly formed with Zn(II) (Figure 21 and Figure 22 and accompanying discussion). Similarly, whereas the structure of [Cd(**17**)_2_]*_n_*·2*n*DMF is a 2D-net (Figure 32d), that of [Cd(**33**)_2_]*_n_*·*n*DMF·2*n*H_2_O is a 3D-network. [Cd(**33**)_2_]*_n_*·*n*DMF·2*n*H_2_O was formed under solvothermal conditions (DMF/H_2_O, 120 °C) from the reaction of Cd(NO_3_)_2_·4H_2_O and H**33**. Interestingly, {Cd_2_(O_2_C)_4_} units assemble (Figure 34a) and bear a resemblance to the 8-connecting dizinc nodes in [Zn(**33**)_2_]*_n_* (Figure 34b, and see also Figure 22b). The 3D-network in [Cd(**33**)_2_]*_n_*·*n*DMF·2*n*H_2_O is described by the authors as having a topological point symbol of {3^3^.4^7^.5^8^.6} [94]. A comparison of the structure of [Cd(**33**)_2_]*_n_*·*n*DMF·2*n*H_2_O (CSD refcode BOBRIW) with that of [Zn(**33**)_2_]*_n_* (refcode TEZJOA) reveals that both compounds crystallize in the monoclinic space group *P*2_1_/*c* with similar cell dimensions (*a* = 21.8088(7), *b* = 10.3071(3), *c* = 29.9710(12) Å, *β* = 126.602(2)° for [Cd(**33**)_2_]*_n_*·*n*DMF·2*n*H_2_O, and *a* = 21.5531(7), *b* = 10.2884(4), *c* = 29.7127(12) Å, *β* = 126.180(2)° for [Zn(**33**)_2_]*_n_*) consistent with very similar assemblies. [Cd(**33**)_2_]*_n_* was shown to be a selective and very sensitive luminescent probe to detect ascorbic acid [94]. The dicarboxylic acid H_2_**37** forms single crystals of [Cd(**37**)(OH_2_)]*_n_*·*n*MeCN·2*n*H_2_O when reacted with H_2_**37** under solvothermal conditions. The [Cd(**37**)(OH_2_)]*_n_* assembly comprises 2-fold interpenetrated 3D-networks and is essentially isostructural with solvated [Zn(**37**)]*_n_* (see Figure 25) [79].

**Figure 35:**
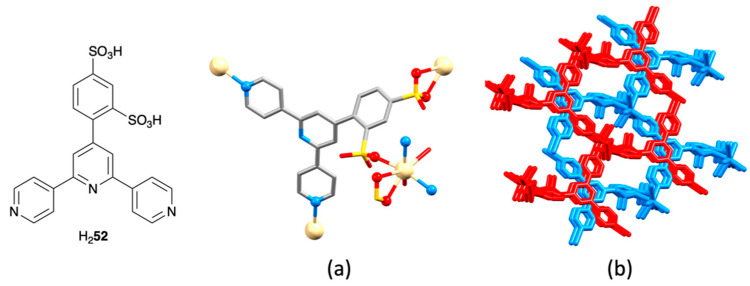
The structure of H_2_**52**. In [Cd(**52**)(OH_2_)_2_]*_n_*, (**a**) both **52**^2–^ and Cd(II) are 4-connecting nodes, and (**b**) the coordination assembly comprises 2-fold, interpenetrating 3D networks (CSD refcodes QJARAU and QJARAU01).

In comparison for carboxylato-functionalized terpyridine ligands, there are far fewer examples bearing sulfonato metal-binding domains. Figure 6 illustrated the 1D-ladder assembly in [Cd(**13**)Cl]*_n_* in which H**13** contains a 2,2′:6′,2″-tpy unit [43]. For the divergent 4,2′:6′,4″-tpy, [Cd(**52**)(OH_2_)_2_]*_n_* (H_2_**52** is defined in Figure 35) is a sole example of a cadmium(II)-containing coordination polymer with a sulfonato-functionalized 4,2′:6′,4″-tpy ligand for which structural data are available. Two independent reports of the synthesis and structure of [Cd(**52**)(OH_2_)_2_]*_n_* appeared in 2015 [95,96]. The compound was prepared under hydrothermal conditions from H_2_**52** and either CdCO_3_ or CdCl_2_·2.5H_2_O, and crystallizes in the triclinic space group *P*–1. As Figure 35a shows, the Cd(II) center is 7-coordinate (distorted pentagonal bipyramidal), being bound by a monodentate SO_3_^–^ group from one **52**^2–^ ligand, a bidentate SO_3_^–^ from a second **52**^2–^, as well as by pyridine rings from two different **52**^2–^ ligands, and two aqua ligands. Both **52**^2–^ and Cd(II) are, therefore, 4-connecting nodes, and the structure propagates into a 3D *dia* network. The large voids in a single net result in 2-fold interpenetration (Figure 35b). Interestingly, when zinc(II) acetate combined with H_2_**52** under the same hydrothermal conditions as for the preparation of [Cd(**52**)(OH_2_)_2_]*_n_*, single crystals of the mononuclear complex [Zn(H**52**)_2_(OH_2_)_4_]·6H_2_O were obtained and PXRD data confirmed that the single crystal was representative of the bulk material [95]. The preference for mononuclear over polymeric assembly is not readily explained.

### 3.9. Assemblies Containing Cadmium(II) and 4,2′:6′,4″-tpy Ligands Functionalized in the 4′-Position with a Pyridinyl Group

**Figure 36:**
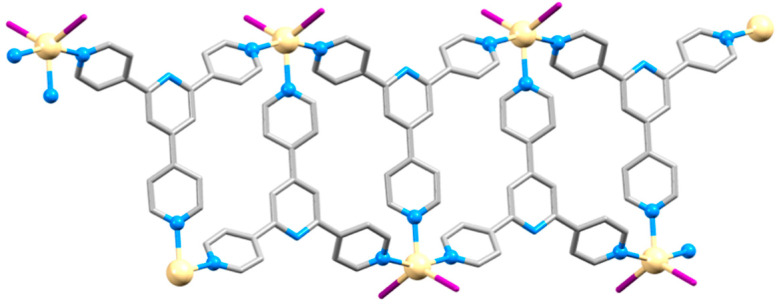
Part of the 1D-ladder in [Cd(**38**)I_2_]*_n_* (DSC refcode USECEC).

Rather surprisingly, there are few examples of structurally characterized coordination assemblies incorporating Cd(II) and 4,2′:6′,4″-tpy ligands with pyridinyl functionalities. Zhang and Li reported the formation of [Cd(**38**)I_2_]*_n_* under hydrothermal conditions (see Scheme 11 for ligand **38**) [97]. The assembly is a 1D-polymer (Figure 36) which may be described as a ladder. Each Cd(II) center is 5-coordinate, and is bound by three different molecules of **38**. The structure is in contrast to the 1D-ladder in [Zn_3_(**39**)_2_Cl_6_]*_n_*·*n*H_2_O (Figure 26b), in which each Zn(II) ion is tetrahedrally sited [82]. Zhang et al. also demonstrated that when the co-ligand benzene-1,4-dicarboxylic acid was added to the CdI_2_/**38** reaction mixture, the role of **38** was reduced to that of a pendant, monodentate ligand and the network was directed purely by benzene-1,4-dicarboxylic acid [97].

### 3.10. Ligands Containing Two 4,2′:6′,4″-tpy Metal-Binding Domains: 4-Connecting Nodes

**Scheme 13:**
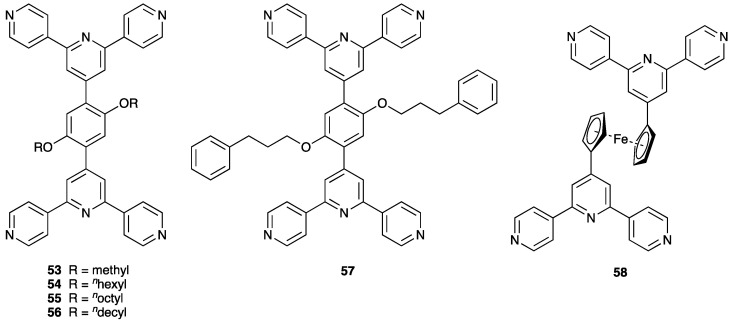
Structures of ligands containing two 4,2′:6′,4″-tpy metal-binding domains.

A strategy for increasing the dimensionality of a coordination assembly is to incorporate ligands that can function as directing nodes. Ditopic ligands such as those in Scheme 9 function only as linkers. In contrast, we have described examples in which the introduction into a 4,2′:6′,4″-tpy unit of a substituent capable of binding a metal ion may allow the ligand to act as a directing node. For example, **36**^2–^ in [Zn(**36**)]*_n_*·4*n*DMF is a 4-connecting node (Figure 25a). Another strategy is to design ligands which possess more than one divergent tpy unit. Examples of ligands which form coordination networks with zinc(II) are displayed in Scheme 13. Crystals of all the zinc(II) complexes described in the section were grown under ambient conditions using layering methods. In an extension of the Hanan one-pot strategy [7] shown in Scheme 3, ligands **53**–**58** were prepared by reaction of an appropriate dialdehyde with four equivalents of 4-acetylpyridine under basic conditions [98,99,100,101].

**Figure 37:**
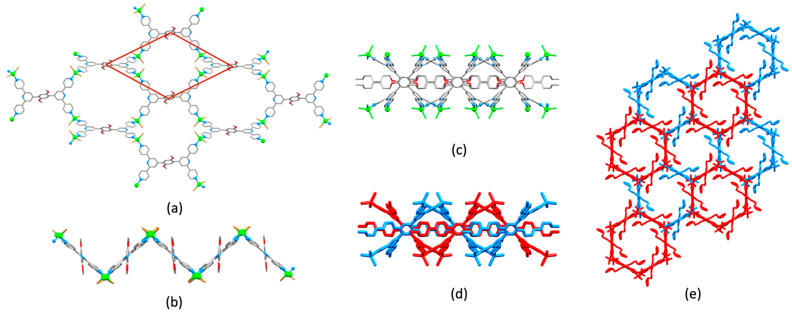
(**a**) Part of the (4,4) network in [Zn_2_Br_4_(**53**)]*_n_*·2*n*C_6_H_4_Cl_2_; each ligand **53** is a 4-connecting node and one rhombus in the net is highlighted in red (CSD refcode ZUGVII). (**b**) A side-view of the 2D-net in [Zn_2_Br_4_(**53**)]*_n_*·2*n*C_6_H_4_Cl_2_. (**c**) In [Zn_2_Cl_4_(**55**)]*_n_*·4*n*H_2_O (refcode NOTPUI), the *n*-octyloxy chains lie in the plane of the network, and (**d**) the same representation of part of one sheet in [Zn_2_Cl_4_(**55**)]*_n_*·4*n*H_2_O showing the 2D→2D parallel interpenetrated nets (red and blue). (**e**) The 2-fold interpenetrating *nbo* nets (red and blue) in [Zn_2_Br_4_(**57**)]*_n_*·*n*H_2_O (refcode DAQMAM).

A combination of **53** and ZnBr_2_ or ZnI_2_ in a 1,2-C_6_H_4_Cl_2_/MeOH solvent system resulted in the growth of crystals of [Zn_2_Br_4_(**53**)]*_n_*·2*n*C_6_H_4_Cl_2_ or [Zn_2_I_4_(**53**)]*_n_*·2.3*n*C_6_H_4_Cl_2_, respectively. Both compounds crystallize in the monoclinic space group *P*2_1_/*n* and are isostructural. Ligand **53** acts as a 4-connecting node and directs the assembly of a 2D-network. The (4,4) net is shown in Figure 37a for [Zn_2_Br_4_(**53**)]*_n_*·2*n*C_6_H_4_Cl_2_. The net has a corrugated profile and the methoxy substituents point above and below the sheet (Figure 37b) [99]. When the *n*-alkyloxy substituent is lengthened to hexyl, octyl or decyl in ligands **54**, **55** or **56**, reactions with zinc(II) chloride or bromide lead to the formation of the 2D→2D parallel interpenetrated networks [Zn_2_Cl_4_(**54**)]*_n_*, [Zn_2_Cl_4_(**55**)]*_n_*·4*n*H_2_O, [Zn_2_Br_4_(**55**)]*_n_*, and [Zn_2_Cl_4_(**56**)]*_n_*·2*n*MeOH [98,99,100,101]. The topology of a single (4,4) net in each compound mimics that in [Zn_2_Br_4_(**53**)]*_n_*·2*n*C_6_H_4_Cl_2_ and [Zn_2_I_4_(**53**)]*_n_*·2.3*n*C_6_H_4_Cl_2_. However, the long alkyloxy chains are directed through the sheet (Figure 37c) and this appears to contribute to the assembly process. Figure 37d illustrates the interpenetrated networks in [Zn_2_Cl_4_(**55**)]*_n_*·4*n*H_2_O [98] and this is representative of the series [Zn_2_Cl_4_(**54**)]*_n_*, [Zn_2_Cl_4_(**55**)]*_n_*·4*n*H_2_O, [Zn_2_Br_4_(**55**)]*_n_*, and [Zn_2_Cl_4_(**56**)]*_n_*·2*n*MeOH. The introduction of a phenyl substituent in a terminal position in the chain as in ligand **57** (Scheme 13) has a profound effect on the assembly process. The reaction between **57** and ZnBr_2_ yielded single crystals of [Zn_2_Br_4_(**57**)]*_n_*·*n*H_2_O. This crystallizes in the trigonal space group *R*–3 with a 3D-architecture, consisting of 2-fold interpenetrating *nbo* nets (Figure 37e). The assembly is directed by the 4-connecting ligand nodes, and the nets are closely associated by virtue of π-stacking between the pendant phenyl rings in **57** and 4,2′:6′,4″-tpy domains in an adjacent net. This results in a highly porous network with a solvent accessible void space of ca. 65% [101].

**Figure 38:**
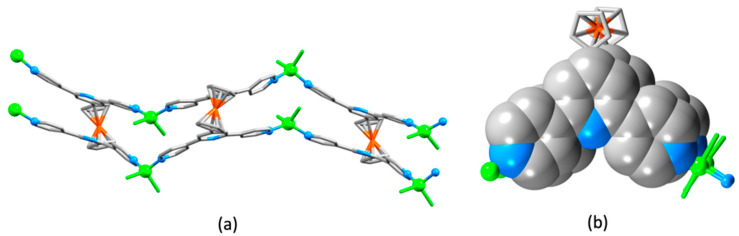
(**a**) Part of 1D-coordination polymer in [ZnCl_2_(**58**)]*_n_*·3*n*CHCl_3_ and (**b**) π-stacking between 4,2′:6′,4″-tpy units in the coordinated ligand **58** (CSD refcode UMUYUY).

Ligand **58** is related to **25** (Scheme 7) but offers two 4,2′:6′,4″-tpy metal-binding domains. However, unlike ligands **53**–**57**, compound **58** exhibits rotational freedom about the ferrocenyl core that allows the ligand to adopt a *cisoid* conformation with the 4,2′:6′,4″-tpy units lying over one another. This impacts on the assembly formed with **58** reacts with ZnCl_2_. In contrast to the networks described above and in Figure 37, [ZnCl_2_(**58**)]*_n_*·3*n*CHCl_3_ is a 1D-coordination polymer (Figure 38a). This appears to be driven by face-to-face π-stacking between 4,2′:6′,4″-tpy units of the same coordinated ligand (Figure 38b) [100].

## 4. 3,2′:6′,3″-Terpyridine

Compared to those involving 4,2′:6′,4″-tpy domains, far fewer coordination assemblies have been reported in which the ligands bear 3,2′:6′,3″-tpy metal binding domains. For those which incorporate zinc(II), we consider them in families of those with coordinatively innocent functionalities attached to the 3,2′:6′,3″-tpy, and those with carboxylate or sulfonate groups which increase the connectivity of the ligand. No structurally characterized zinc(II)-containing assemblies with unfunctionalized 3,2′:6′,3″-tpy or pyridinyl-substituted 3,2′:6′,3″-tpy ligands had been reported in the CSD at the time of writing this review.

In this section, we provide schematic representations of the conformations of the coordinated 3,2′:6′,3″-tpy units to emphasize its flexibility. Note, however, that in these diagrams, we use the three limiting, planar conformations.

### 4.1. Assemblies Containing Zinc(II) and 4′-Functionalized 3,2′:6′,3″-tpy Ligands with Coordinatively Innocent Substituents

**Figure 39:**
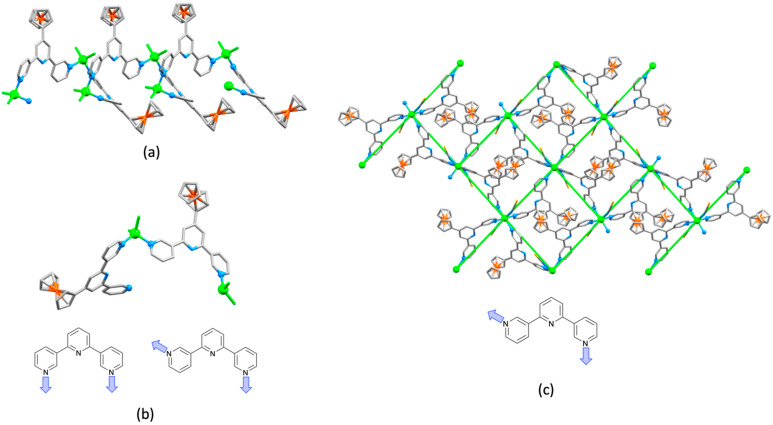
(**a**) Part of the 1D-polymer in [Zn_2_Cl_4_(**59**)_2_]*_n_*·3*n*H_2_O (CSD refcode VUKMOF) and (**b**) the asymmetric unit in [Zn_2_Cl_4_(**59**)_2_]*_n_*·3*n*H_2_O and diagram showing the different conformations (in planar notation) of the crystallographically independent ligands **59**. (**c**) Part of the (4,4) net observed in [Zn_3_(NCS)_6_(**59**)_6_]·6*n*CHCl_3_ (refcode OGOYIU). Each Zn is a 4-connecting node and the Zn_4_ rhombuses are highlighted; the conformation of the 3,2′:6′,3′′-tpy units is shown in planar notation.

In Section 3.2, we described competition between the formation of polymeric and discrete molecular assemblies when zinc(II) salts reacted with 1-(4,2′:6′,4′′-terpyridin-4′-yl)ferrocene (**25**, Scheme 7). Under conditions of crystal growth by layering, the isomer of **25**, 1-(3,2′:6′,3′′-terpyridin-4′-yl)ferrocene (**59**) combines with ZnCl_2_ to yield single crystals of the discrete metallosquare [Zn_4_Cl_8_(**59**)_4_]·3CHCl_3_·3MeOH [57]. However, only a few crystals were obtained, and the bulk material was an amorphous powder for which PXRD data could not be obtained. Significantly, Xiao and Tian reported the formation of the 1D-polymer [Zn_2_Cl_4_(**59**)_2_]*_n_*·3*n*H_2_O (Figure 39a), but no synthetic details are available [102]. However, this observation provides support for possible competition between molecular and polymer assembly. [Zn_2_Cl_4_(**59**)_2_]*_n_*·3*n*H_2_O crystallizes in the triclinic space group *P*–1 and the chain is built up by translation. The asymmetric unit contains two crystallographically independent ligands which differ in their conformations as shown in Figure 39b. This demonstrates the conformational flexibility of the 3,2′:6′,3′′-tpy unit (see Scheme 4), which sets it apart from the isomeric 4,2′:6′,4′′-tpy. We have also shown that the reaction of ZnBr_2_ and **59** yielded [Zn_2_Br_4_(**59**)_2_]*_n_*·2*n*MeOH (CSD refcode TAYSIY) which has a very similar polymeric structure [57] to [Zn_2_Cl_4_(**59**)_2_]*_n_*·3*n*H_2_O. Xiao and Tian [103] have reported [Zn_2_I_4_(**59**)_2_]*_n_* which is structurally analogous to the chlorido and bromido complexes. Changing to zinc(II) thiocyanate has the effect of switching the assembly with ligand **59** to a 2D-network. The structure of [Zn_3_(NCS)_6_(**59**)_6_]·6*n*CHCl_3_ is a communication to the CSD (refcode OGOYIU [104]). Each Zn center is bound by four pyridine rings from four different ligands **59**, and the octahedral coordination sphere is completed by two thiocyanato ligands in a *trans* arrangement. The resulting (4,4) net is shown in Figure 39c. The asymmetric unit contains three independent molecules of **59** but all the 3,2′:6′,3′′-tpy units have similar conformations (Figure 39c).

### 4.2. Assemblies Containing Zinc(II) and 3,2′:6′,3″-tpy Ligands Functionalized with Carboxylate or Sulfonate Donors

**Scheme 14:**
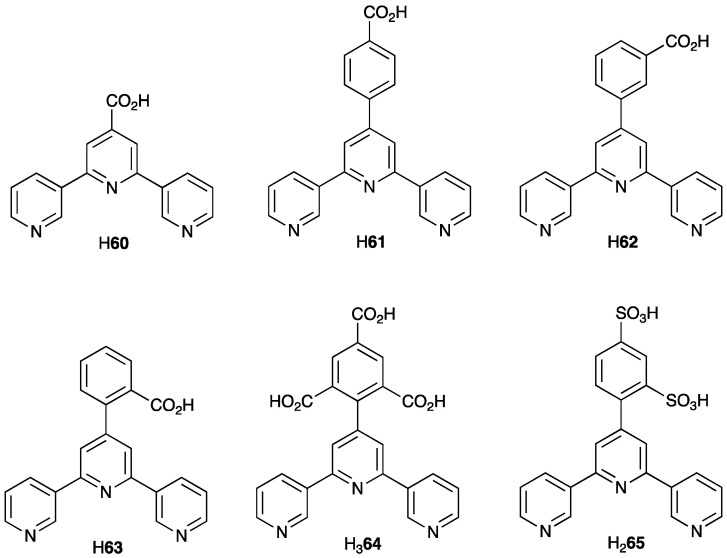
Structures of 3,2′:6′,3″-tpy ligands functionalized with carboxylic or sulfonic acid groups.

The structures of the ligands relevant to this section are depicted in Scheme 14. As has already been discussed, the 3,2′:6′,3″-tpy metal-binding domain is conformationally flexible and only one planar conformation is shown for each ligand in Scheme 14. Crystals of zinc(II) complexes incorporating H**60**–H**63**, H_3_**64**, and H_2_**65** described in this section were grown under solvothermal conditions.

**Figure 40:**
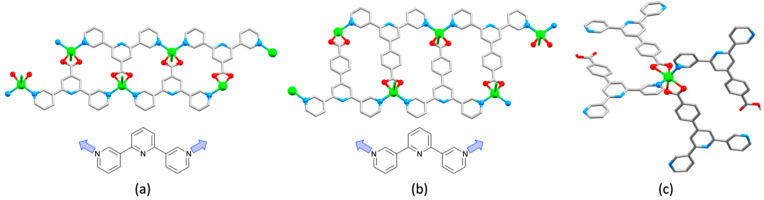
Part of the 1D-polymer in (**a**) Zn_2_Cl_2_(**60**)_2_]*_n_* (CSD refcode XEWGUD), and (**b**) [ZnCl(**61**)]*_n_* (CSD refcode FIJJIP); the conformation of the 3,2′:6′,3′′-tpy unit is shown in planar notation for both structures. (**c**) The structure of the mononuclear complex [Zn(**61**)_2_(H**61**)_2_] in the dihydrate (refcode FEJJEL); H atoms except for those in the CO_2_H groups are omitted.

**Figure 41:**
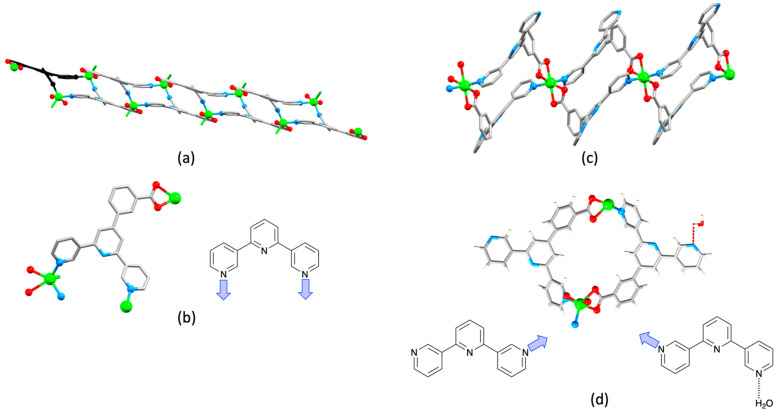
(**a**) Part of the ‘double-decker′ 2D-network in [ZnCl(**62**)]*_n_* (CSD refcode IKEXED); one **62**^–^ ligand is shown in black to emphasize the conformation of the ligand. (**b**) The building block in [ZnCl(**62**)]*_n_* and the planar conformation of the 3,2′:6′,3″-tpy unit. (**c**) Part of the loop-like 1D-polymer in [Zn(**62**)_2_]*_n_·*2*n*H_2_O (refcode IKEXIH), and (**d**) detail of one loop in the chain showing two different ligand conformations; one non-coordinated pyridine ring is involved in hydrogen bond formation.

Hu and coworkers have reported that a combination of H**60** with ZnCl_2_ under hydrothermal conditions (pH 4.0, 180 °C) produced [Zn_2_Cl_2_(**60**)_2_]*_n_* which is a 1D-coordination polymer in which each **60**^–^ binds to three Zn(II) centers [105]. Note that the conformational flexibility of the 3,2′:6′,3″-tpy unit facilitates this ladder-like assembly (Figure 40a) and should be contrasted with the V-shaped building block offered by the 4,2′:6′,4″-tpy unit in H**31** (an isomer of H**60**) and discussed in Section 3.4. In related work, Hu and coworkers used H**61** in reactions with ZnCl_2_. Depending upon the pH, either [Zn(**61**)_2_(H**61**)_2_]·2*n*H_2_O (at pH 4, see below) or [ZnCl(**61**)]*_n_* (at pH 5) was produced under hydrothermal conditions in the presence of 1,10-phenanthroline (phen) at 180 °C [106]. It appears that phen plays a templating role; other examples in which template seemed to be required were described earlier, and we see a further example with ligand H**62** (see below). The preparation and structure of [ZnCl(**61**)]*_n_* has also been reported by Zhang et al. (CSD refcode FIJJIP01) [107]. As in [Zn_2_Cl_2_(**60**)_2_]*_n_* (Figure 40a), a 1D-polymer is observed in [ZnCl(**61**)]*_n_* and Figure 40b illustrates that the 3,2′:6′,3″-tpy unit adopts a common conformation in both polymers. In the investigation from Hu [106], the product [Zn(**61**)_2_(H**61**)_2_]·2*n*H_2_O formed at pH 4 is a discrete molecular complex. The Zn(II) center is bound by two monodentate H**61** ligands and two **61**^–^ coordinated through bidentate carboxylate groups (Figure 40c). The conformation of the 3,2′:6′,3″-tpy unit is the same as in the 1D-polymers in Figure 40a,b. Since H**61** is an isomer of H**17** and both offer divergent *N*-donor sets, it is instructive to compare their reactions with ZnCl_2_. As we detailed earlier, combinations of ZnCl_2_ and H**17** have led to [Zn_2_(H**17**)Cl_2_]*_n_*·2*n*EtOH·*n*H_2_O (Figure 14) [48], and [Zn_2_(**17**)_2_Cl_2_]*_n_*·0.5*n*H_2_O (a 2-fold interpenetrated 6^3^ net) [70]. Carboxylic acids H**62** and H**63** are isomers of H**61** (Scheme 14), and a comparison can be made between the reactions of H**61** or H**62** with ZnCl_2_ (all under solvothermal conditions). A combination of ZnCl_2_ and H**62** (pH 6, 180 °C) resulted in single crystals of [ZnCl(**62**)]*_n_*which possesses a 2D-network with each Zn(II) center and each **62**^–^ ligand acting as a 3-connecting node [108]. Figure 41a shows a side-view of the 2D-network, and illustrates how the conformation of the ligand facilitates the assembly of the double-decker type architecture; the angles between the planes of pairs of connected pyridine rings are 19.3 and 37.7°. The building block in the network is shown in Figure 41b, with a schematic representation of the conformation of the 3,2′:6′,3″-tpy unit. Hu and coworkers also carried out the reaction of H**62** and ZnCl_2_ in the presence of malonic acid. The latter does not act as a co-ligand and the assembly is another example of a possible templating effect. Crystals of [Zn(**62**)_2_]*_n_·*2*n*H_2_O were obtained and structural characterization revealed a 1D-coordination polymer with a loop-like architecture (Figure 41c). Each pair of adjacent Zn(II) centers is bridged by two ligands **62**^–^, each ligand coordinating through a bidentate CO_2_^–^ group and one pyridine *N*-donor. Although not discussed in the original work [108], inspection of the packing interactions in the structure (CSD refcode IKEXIH) indicates that hydrogen bonding from one non-coordinated N_py_ atom to a water molecule may be responsible for the different conformations of the 3,2′:6′,3″-tpy units (Figure 41d).

**Figure 42:**
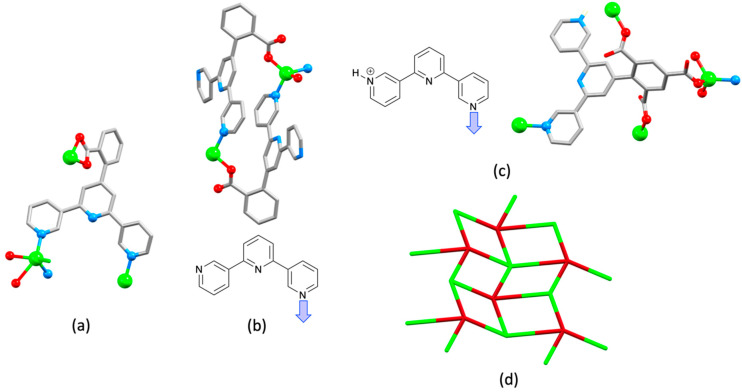
(**a**) The building block in [ZnCl(**63**)]*_n_·n*H_2_O (CSD refcode MICTAW); compare this with Figure 41b. (**b**) Detail of one loop in the 1D-chain in [Zn(**63**)_2_]*_n_* (refcode MICTEA) and the ligand conformation; one terminal pyridine ring is non-coordinated; compare with Figure 41d. (**c**) The building block in [Zn(H**64**)]*_n_*·1.5*n*H_2_O (refcode MUNDEH) and ligand conformation. (**d**) Part of the (4,4) net in [Zn(H**64**)]*_n_*·1.5*n*H_2_O with 4-connecting Zn nodes (green) and ligand nodes (red); the ligand node is defined as the centroid of the central pyridine ring.

On going from H**61** and H**62** to H**63** (Scheme 14), there are no data available to directly compare reactions of ZnCl_2_ with the final isomer in the series. However, crystals of [ZnCl(**63**)]*_n_·n*H_2_O were prepared in the hydrothermal reaction of H**63** and Zn(OAc)_2_ with the addition of EuCl_3_·6H_2_O and glutaric acid (pH 3.5, 180 °C). The assembly in [ZnCl(**63**)]*_n_·n*H_2_O is unlike either of those in [ZnCl(**61**)]*_n_* [106,107] or [ZnCl(**62**)]*_n_* [109]. In [ZnCl(**63**)]*_n_·n*H_2_O, each Zn(II) is bound by two *N*-donors of two different **63**^–^ ligands, a bidentate CO_2_^–^ from a third **63**^–^ ligand, and a chlorido ligand. Each Zn(II) and each **63**^–^ is a 3-connecting node and the assembly propagates into a 3D-network with a *utp* topology [11]. Note that the building block in [ZnCl(**63**)]*_n_·n*H_2_O (Figure 42a) is similar to that in [ZnCl(**62**)]*_n_* (Figure 41b) and adopts an analogous conformation of the 3,2′:6′,3″-tpy unit. Hu and coworkers have also reported the single crystal structure of [Zn(**63**)_2_]*_n_* [11] and this exhibits a similar loop-like 1D-coordination polymer as observed in [Zn(**62**)_2_]*_n_·*2*n*H_2_O (see above). However, a comparison of Figure 42b with Figure 41d reveals a ‘tighter′ loop in [Zn(**63**)_2_]*_n_* than in [Zn(**62**)_2_]*_n_·*2*n*H_2_O, as well as a change in the conformation of the 3,2′:6′,3″-tpy unit.

The tricarboxylic acid H_3_**64** (Scheme 14) has been combined with Zn(NO_3_)_2_·6H_2_O under hydrothermal conditions with phen added to the reaction mixture. As with many of the investigations reviewed in this section, the aim was to prepare a fluorescent material for sensing applications. Crystals of [Zn(H**64**)]*_n_*·1.5*n*H_2_O (CSD refcode MUNDEH) were isolated, and contain H**64**^2–^ in which one outer pyridine ring bears the proton. The asymmetric unit with symmetry-generated atoms is shown in Figure 42c. Both H**64**^2–^ and Zn(II) are 4-connecting nodes, and a 2D (4,4)-net (Figure 42d) is assembled [109].

**Figure 43:**
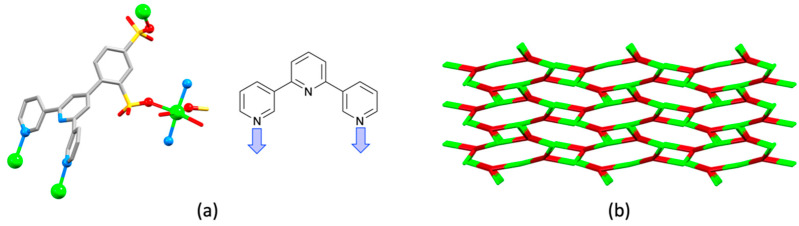
(**a**) The building block and representation of the conformation of the 3,2′:6′,3″-tpy unit in [Zn(**65**)(OH_2_)_2_]*_n_*·2*n*H_2_O (CSD refcode DUWDUW), and (**b**) part of the 3D *sar* network in [Zn(**65**)(OH_2_)_2_]*_n_*·2*n*H_2_O with 4-connecting Zn nodes (green) and 4-connecting ligand nodes (red); the ligand node is defined as the centroid of the central pyridine ring.

Finally in this section, we consider the assembly formed when Zn(NO_3_)_2_·6H_2_O was treated with the disulfonic acid H_2_**65** (Scheme 14) under hydrothermal conditions (pH 7, 120 °C). Crystals of [Zn(**65**)(OH_2_)_2_]*_n_*·2*n*H_2_O were isolated in 78% yield, and the coordination compound is isostructural with that of the cadmium(II) analog [110]. Ligand **65**^2–^ is a 4-connecting node as shown in Figure 43a, and each Zn(II) center is octahedrally sited, bound by two pyridine *N*-donors in a *trans*-arrangement, two monodentate sulfonato groups (*cis* with respect to one another) from two different **65**^2–^ ligands, and two aqua ligands. Like ligand **65**^2–^, the Zn(II) center is a 4-connecting node, and a 3D-network with a *sar* topology is produced (Figure 43b).

### 4.3. Assemblies Containing Cadmium(II) and 3,2′:6′,3″-tpy Ligands

**Figure 44:**
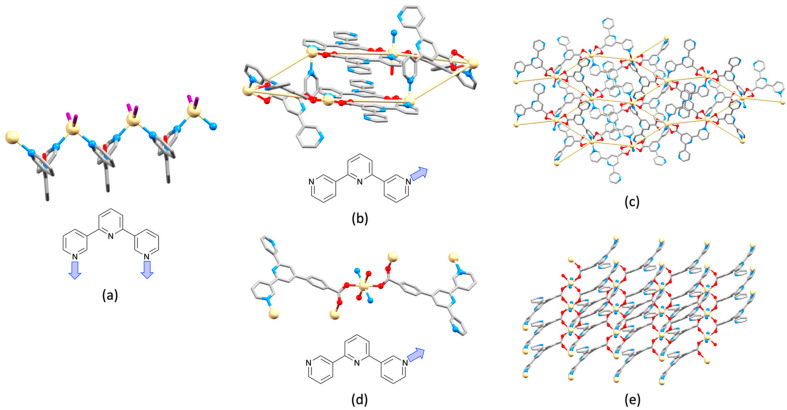
(**a**) Part of the 1D-polymer in [CdI_2_(**66**)]*_n_* (CSD refcode HATFOA), and the limiting planar conformation. (**b**) Part of one 6-membered circuit in the (6,3) net in [Cd(**60**)_2_(OH_2_)]*_n_* (refcode XEWGEN); there are two crystallographically independent **60**^–^ ligands but both can be described by the same limiting planar conformation. (**c**) Part of the (6,3) net in [Cd(**60**)_2_(OH_2_)]*_n_*. (**d**) The 6-connecting Cd(II) and 3-connecting ligand nodes in [Cd(**61**)_2_]*_n_* with a schematic diagram of the planar conformation of the 3,2′:6′,3″-tpy, and (**e**) part of the 2D-network in [Cd(**61**)_2_]*_n_* (refcode DOGFEN) viewed down the *c*-axis.

Only a few examples of coordination polymers and networks comprising Cd(II) and 3,2′:6′,3″-tpy ligands have been described. With one exception (CSD refcode HATFOA, see below), in all cases, the 3,2′:6′,3″-tpy is functionalized in the 4′-position with either a carboxylic or sulfonic acid. The exception is the ligand [3,2′:6′,3′′-terpyridine] *N*′-oxide (**66**) which was combined with CdI_2_ under conditions of crystal growth by layering. The interest in using an *N*-oxide was to incorporate catalytically active species into solid coordination polymer frameworks [111]. Crystals of the 1D-coordination polymer [CdI_2_(**66**)]*_n_* were isolated. The polymer chain (Figure 44a) is built up by a 2-fold rotation axis and equal numbers of left- and right-handed helical chains are present in the crystal lattice. The angle between the planes of the middle and terminal pyridine rings is 43.3°, and Figure 44a shows the most appropriate limiting planar conformation of the 3,2′:6′,3″-tpy unit. Dong and coworkers also confirmed that [HgI_2_(**66**)]*_n_* is isostructural with [CdI_2_(**66**)]*_n_*. These and related coordination polymers (see Section 5) were shown to be heterogeneous catalysts for Knoevenagel condensation reactions under ambient conditions [111].

**Figure 45:**
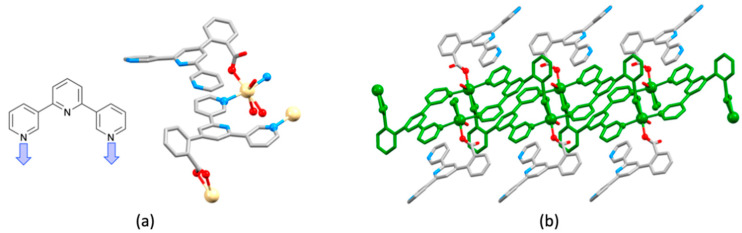
The structure of [Cd(**63**)_2_(OH_2_)]*_n_* (CSD refcode MICTOK). (**a**) The asymmetric unit with symmetry generated atoms, and the planar conformation of both independent 3,2′:6′,3″-tpy units. (**b**) A representation of part of the 1D-polymer illustrating the different roles of the two independent **63**^–^ ligands; the ligands colored green comprise the polymer backbone, while the second ligands are peripheral to the assembly.

Zinc(II) coordination polymers and networks involving carboxylic and sulfonic acid functionalized ligands H**60**, H**61**, H**63** and H_2_**65** (Scheme 14) were discussed in Section 4.2. We now compare the effects of moving from Zn(II) to Cd(II). For H**60**, a direct comparison between Zn and Cd cannot be made since H**60** has been combined with zinc(II) chloride (hydrothermal conditions, pH 4, 180 °C) as opposed to cadmium(II) nitrate (hydrothermal, pH 5, 180 °C) [105]. In the latter reaction, crystals of [Cd(**60**)_2_(OH_2_)]*_n_* were obtained. The Cd(II) center is 7-coordinate, being bound by two bidentate carboxylates from two different ligands, by two terminal pyridine rings of two additional ligands, and by an aqua ligand. The remaining two *N*-donors of each ligand are uncoordinated. The assembly of a 2D-network is thereby directed by the Cd(II) nodes, and each **60**^–^ ligand serves as a linker. In contrast to the (4,4) net in [Cd_2_(**34**)_4_]*_n_*·*n*H_2_O (see Figure 33) in which each Cd(II) is 4-connecting and each Cd…Cd vector is bridged by one ligand, the assembly in [Cd(**60**)_2_(OH_2_)]*_n_* is a (6,3) net in which Cd(II) is a 3-connecting node. Two of the six Cd…Cd vectors in the 6-membered circuit are each bridged by two **60**^–^ ligands, and four Cd…Cd vectors are each bridged by one ligand (Figure 44b). Part of the 2D-network is depicted in Figure 44c [105]. Going from H**60** to H**61** introduces a phenylene spacer, and under solvothermal conditions (MeCN/H_2_O, 160 °C), the reaction of H**61** and Cd(NO_3_)_2_·6H_2_O leads to crystals of [Cd(**61**)_2_]*_n_*. The Cd(II) center lies on a center-of-symmetry and is 6-coordinate, and bound to six different ligands (Figure 44d). Given the availability of both pyridine and carboxylato donors, we again see the preference for CO_2_^–^ coordination to Cd(II), and the 3,2′:6′,3″-tpy domain retains two uncoordinated pyridine rings and adopts the conformation shown in Figure 44d. The combination of 6-connecting Cd(II) and 3-connecting **61**^–^ ligand leads to the 2D-network shown in Figure 44e. A noteworthy feature of the 2D-sheet is the incorporation of infinite {Cd_2_(μ-O_2_CR)_2_}*_n_* chains. [Cd(**61**)_2_]*_n_* is strongly luminescent and its application as a luminescence sensor for 2,4,6-trinitrophenol was demonstrated [112]. When H**63** was combined with CdCl_2_·2.5H_2_O under hydrothermal conditions (pH 6, 180 °C) and in the presence of benzene-1,2-dicarboxylic acid, crystals of [Cd(**63**)_2_(OH_2_)_2_]*_n_* (CSD refcode MICTIE) were isolated. The structure is a loop-like 1D-polymer [11], similar to that found in [Zn(**63**)_2_]*_n_* (Figure 42b). The major difference is the incorporation of two aqua ligands per Cd(II), consistent with the preference for a higher coordination number for the larger metal ion. A change in the pH from 6.0 to 4.0 resulted in the assembly of [Cd(**63**)_2_(OH_2_)]*_n_* which is again a 1D-polymer comprising loop-like building blocks [11]. The structure is rather unusual. [Cd(**63**)_2_(OH_2_)]*_n_*crystallizes in the monoclinic space group *P*2_1_/*c* and Figure 45a shows the asymmetric unit with symmetry generated atoms. There are two independent **63**^–^ ligands, and the two different 3,2′:6′,3″-tpy units adopt the same conformation. However, while one 3,2′:6′,3″-tpy coordinates to two Cd(II) centers, the second is non-coordinated and simply decorates the periphery of the chain (Figure 45b). The assembly in [Cd(**63**)_2_(OH_2_)]*_n_* once again illustrates the dominance of the anionic carboxylato donor over coordination through the terpyridine domain. Finally, for the sulfonato-functionalized ligand **65**^2–^, [Cd(**65**)(OH_2_)_2_]*_n_*·2*n*H_2_O (CSD refcode DUWFAE) is isostructural with [Zn(**65**)(OH_2_)_2_]*_n_*·2*n*H_2_O (Figure 43) [110].

## 5. 4,3′:5′,4″-Terpyridine

**Scheme 15:**
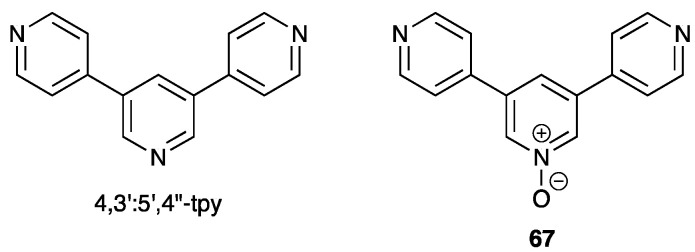
The structure of 4,3′:5′,4″-terpyridine and of the *N*-oxide **67**.

Like 4,2′:6′,4″-tpy, the isomer 4,3′:5′,4″-tpy offers a V-shaped building block for coordination polymer assembly. However, this ligand has been little utilized, and coordination polymers involving Zn(II) and Cd(II) are limited to those directed by ligand **67** (Scheme 15), plus one example which also involves a co-ligand [113].

**Figure 46:**
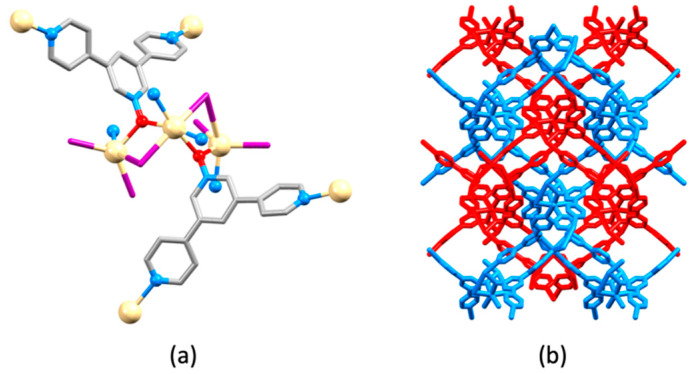
The structure of [Cd_3_I_6_(**67**)_2_]*_n_* (CSD refcode HATFIU). (**a**) The building block in the 3D-assembly showing the {Cd_3_I_4_(μ-I)_2_(μ-O)_2_N_4_} unit, and (**b**) part of the 2-fold interpenetrating 3D network view down the crystallographic *a*-axis (terminal I atoms are omitted).

As discussed in Section 4.3, Dong and coworkers have been interested in incorporating catalytically active species in the form of terpyridine *N*-oxides into coordination polymer frameworks [111]. In addition to using [3,2′:6′,3′′-terpyridine] *N*′-oxide (**66**), they have reported the reactions of ZnCl_2_ and ZnI_2_ with [4,3′:5′,4′′-terpyridine] *N*′-oxide (**67**). Crystal growth was under ambient conditions, and the structurally similar 1D-coordination polymers [ZnCl_2_(**67**)]*_n_* and [ZnI_2_(**67**)]*_n_* were formed (CSD refcodes HATFAM and HATFEQ). Both contain helical chains which are built up by a screw axis. In contrast, the reaction of CdI_2_ with **67** (carried out under similar layering conditions to ZnI_2_ with **67**) gave crystals of [Cd_3_I_6_(**67**)_2_]*_n_* which crystallizes in the tetragonal space group *I*4_1_/*a*. Both the outer pyridine rings of **67** and the *N-*oxide bind to Cd(II). Trinuclear {Cd_3_I_4_(μ-I)_2_(μ-O)_2_N_4_} units assemble (Figure 46a). Ligand **67** acts as a 3-connecting node, and the central Cd(II) of the Cd_3_ cluster is a 4-connecting node. The structure propagates into the 2-fold interpenetrating 3D network shown in Figure 46b [111].

Given the relative rigidity of 4,3′:5′,4″-tpy, and the fact that there is precedent for all three pyridine rings to be involved in coordination [114,115], there is significant scope for further exploration of this ligand in coordination polymer assemblies.

## 6. Conclusions

In this review, we have presented a survey of coordination polymers and networks containing isomers of terpyridine combined with zinc(II) or cadmium(II). Although 48 isomers of terpyridine can be drawn, three dominate the coordination chemistry of Zn(II) and Cd(II) in infinite assemblies. These are 2,2′:6′,2″-tpy, 4,2′:6′,4″-tpy, and 3,2′:6′,3″-tpy. A few examples utilizing 4,3′:5′,4″-tpy or its *N*′-oxide have also been described. For 2,2′:6′,2″-tpy, the propensity for chelation means that extended assemblies rely on the introduction of substituents that incorporate additional coordination sites. All of the coordination polymers containing Zn(II) or Cd(II) with 2,2′:6′,2″-tpy involve {M(2,2′:6′,2″-tpy)X*_n_*} domains, and there are no examples containing {M(2,2′:6′,2″-tpy)_2_} cores. Both 4,2′:6′,4″-tpy, and 3,2′:6′,3″-tpy can be classed as divergent ligands, and there is a wide range of extended structures incorporating these ligands bound to Zn(II) or Cd(II). The addition of functionalities such as carboxylic acid or pyridinyl units has led to a wealth of different networks, but predictive crystal engineering in these systems is difficult. What is clear, however, is that the presence of an anionic carboxylate donor in functionalized 4,2′:6′,4″-tpy and 3,2′:6′,3″-tpy ligands often leads to preferential coordination by the CO_2_^–^ to Zn(II) or Cd(II), with the result that a significant number of assemblies exhibit pendant, non-coordinated pyridine units.

Our focus has been on structural data. Where possible, we have demonstrated the effects of going from Zn(II) to the larger Cd(II) while retaining the same ligand. However, in trying to make such comparisons, we have noted a significant lack of systematic investigations, and without such studies, it is difficult to even begin to understand why a particular assembly arises from a given combination of metal ion and terpyridine ligand.

We have also highlighted cases where there is structural evidence for the competitive assembly of different products, for example, competition between the formation of a 1D-polymer and a metallomacrocycle. In these cases, in particular, the need for PXRD analysis of bulk materials is essential but is, often, lacking. Indeed, since coordination polymer chemistry typically involves the growth of single crystals, the selection of one crystal, and a structure determination, bulk sample characterization is essential if one is to be able to say anything about the significance of the singe crystal structure that is presented.

## Figures and Tables

**Table 1:** 1D-coordination polymers of general formula [Zn(4′-X-4,2′:6′,4″-tpy)Cl_2_]*_n_*.

Compound	CSD Refcode	Space Group	N–Zn–N/°	Zn…Zn/Å	Ref.
[Zn(4,2′:6′,4″-tpy)Cl_2_]*_n_*	GAQYUS	*P*2_1_/*n*	98.6(2)	12.528(4)	[44]
[Zn(**14**)Cl_2_]*_n_*	FEPRUO	*P*2_1_/*n*	105.96(11)	13.040(1)	[46]
[Zn(**15**)Cl_2_]*_n_*	LOCTED	*P*2_1_/*n*	99.5(1)	12.992(1)	[47]
[Zn(**15**)Cl_2_]*_n_* ^a^	NOGFIZ	*P*3_1_21	107.9; 100.3(2)	12.564; 13.029(2)	[47]
[Zn(**16**)Cl_2_]*_n_*·*n*EtOH·*^n^*/_2_H_2_O	NOGFOF	*P*2_1_/*n*	93.5(1)	12.379(2)	[47]
[Zn_2_(H**17**)Cl_2_]*_n_*·2*n*EtOH·*n*H_2_O	DIRMEY	*Pnna*	101.1; 97.6	12.626	[48]
[Zn(**18**)Cl_2_]*_n_*	DOMBIS	*P*2_1_/*n*	100.06(1)	13.037(1)	[49]
[Zn(**19**)Cl_2_]*_n_*·*n*H_2_O	SEBRID	*Pna*2_1_	100.3(2)	12.706(1)	[50]
[Zn(**20**)Cl_2_]*_n_*	AJURIG	*P*2_1_/*c*	104.01(10)	12.8502(6)	[51]
[Zn(**21**)Cl_2_]*_n_*	ELAMOV	*P*2_1_/*c*	103.88(8)	12.523(2)	[14]
[Zn(**22**)Cl_2_]*_n_*	QOJBOI	*P–*1	97.4(1), 98.1(1), 102.8(1), 104.4(1)	12.400(1), 12.325(2), 12.863(2)	[52]
[Zn(**23**)Cl_2_]*_n_*	QOJBAU	*P*–1	104.6(1), 94.3(2), 100.2(1), 103.0(1)	12.899(1), 12.4525(9), 13.062(1), 12.3425(9)	[52]

^a^ Homochiral.

**Table 2:** 1D-coordination polymers of general formula [Zn(4′-X-4,2′:6′,4″-tpy)X_2_]*_n_* with X^–^ = I^–^, AcO^–^, NCS^–^.

Compound	CSD Refcode	Space Group	N–Zn–N/°	Zn…Zn/Å	Ref.
[Zn(**14**)(OAc)_2_]*_n_*	CUXDOP	*P*2_1_/*n*	106.99(5)	12.583(2)	[54]
[Zn(**21**)I_2_]*_n_*·*n*H_2_O	ELAMEL	*P*2_1_/*c*	98.1(1)	12.583(2)	[14]
[Zn(**24**)I_2_]*_n_*·*n*C_6_H_4_Cl_2_	FAKRUG	*P*2_1_/*c*	105.0(1)	12.6858(7)	[55]
[Zn(**25**)I_2_]*_n_*·2*n*CHCl_3_	TUNKEU	*P*2_1_/*c*	104.5(1)	12.771(2)	[56]
[Zn(**25**)(OAc)_2_]*_n_*·2*n*H_2_O	TUNCIQ	*P*2_1_/*n*	106.8(2)	12.516(5)	[56]
[Zn(**25**)(NCS)_2_]*_n_*·*n*CHCl_3_	TUNNIB	*P*2_1_/*n*	111.7(1) ^a^	12.416(3)	[56]
[Zn(**25**)(OAc)_2_]*_n_*·*n*MeOH·*n*H_2_O	NEFWED	*Pbca*	110.06(9)	12.1240(9)	[57]
(*M*)-[Zn(**26**)(OAc)_2_]*_n_*	OFIVOQ	*P*2_1_2_1_2_1_	103.2(2)	12.5757(9)	[58]
[2{Zn(**26**)(OAc)_2_}]*_n_*·*n*CHCl_3_·5*n*MeOH·2*n*H_2_O	OFIWEH	*Pnna*	114.29	13.194	[58]

^a^ N–Zn–N angle within the polymer chain.

## Data Availability

There are no origibal data available for this review article.

## Supplementary Materials

The following is available online: Figure S1: Structures of the 47 isomers of terpyridine other than 2,2′:6′,2″-tpy.
