# Supplementary material for: Isomers of Terpyridine as Ligands in Coordination Polymers and Networks Containing Zinc(II) and Cadmium(II)
Source: Molecules. 2021 May 23;26(11):3110. doi: 10.3390/molecules26113110 (PMC8197025; doi:10.3390/molecules26113110)
Supplement: Supplementary file 1 [file molecules-26-03110-s001.zip › molecules-1226161-supplementary.pdf]

Supporting information

Isomers of terpyridine as ligands in coordination polymers and networks containing zinc(II) and cadmium(II)

Catherine E. Housecroft *, Edwin C. Constable
University of Basel, Department of Chemistry, BPR 1096, Mattenstrasse 24a, CH-4058 Basel, Switzerland

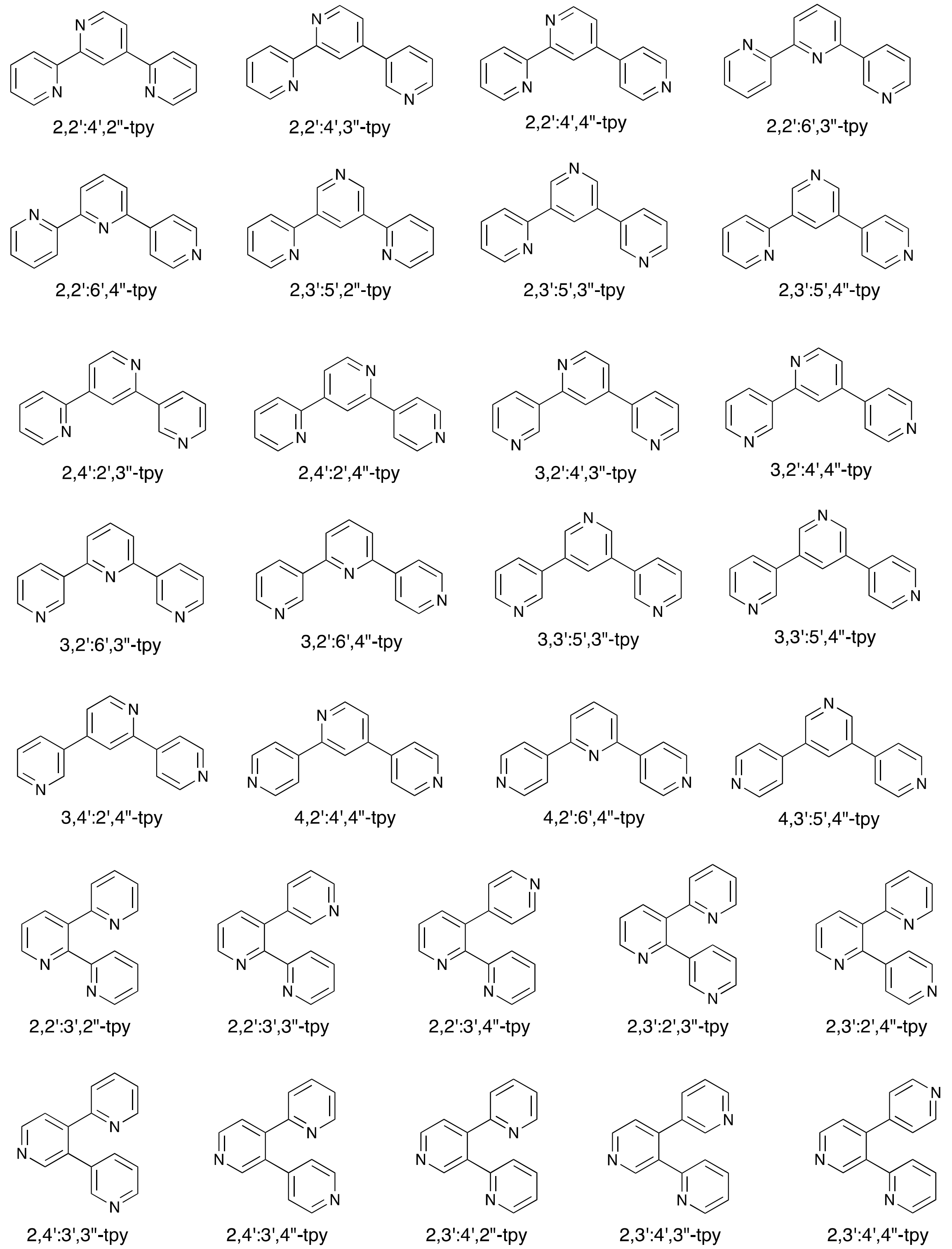

Figure S1. (part 1) The 47 isomers of terpyridine other than 2,2':6',2"-tpy.

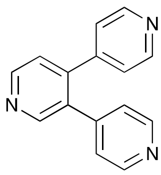

4,3':4',4"-tpy

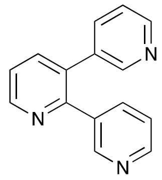

3,2':3',3"-tpy

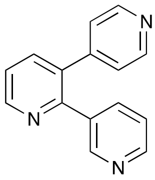

3,2':3',4"-tpy

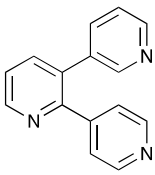

3,3':2',4"-tpy

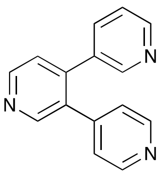

3,4':3',4"-tpy

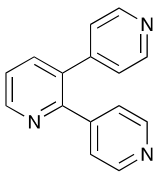

4,2':3',4"-tpy

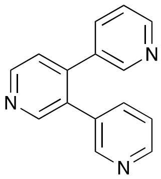

3,3':4',3"-tpy

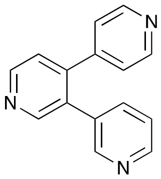

3,3':4',4"-tpy

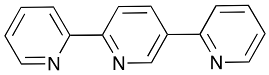

2,2':5',2"-tpy

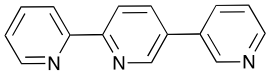

2,2':5',3"-tpy

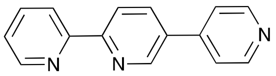

2,2':5',4"-tpy

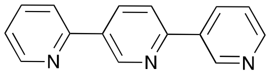

2,3':6',3"-tpy

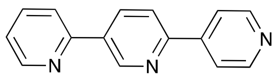

2,3':6',4"-tpy

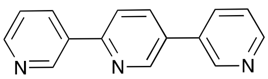

3,2:5',3"-tpy

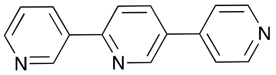

3,2':5',4"-tpy

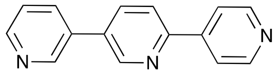

3,3':6',4"-tpy

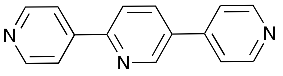

4,2':5',4"-tpy

Figure S1. (part 2) The 47 isomers of terpyridine other than 2,2':6',2"-tpy.
